# Data quality and patient characteristics in European ANCA-associated vasculitis registries: data retrieval by federated querying

**DOI:** 10.1136/ard-2023-224571

**Published:** 2023-10-31

**Authors:** Karl Gisslander, Matthew Rutherford, Louis Aslett, Neil Basu, François Dradin, Lucy Hederman, Zdenka Hruskova, Hicham Kardaoui, Peter Lamprecht, Sabina Lichołai, Jacek Musial, Declan O’Sullivan, Xavier Puechal, Jennifer Scott, Mårten Segelmark, Richard Straka, Benjamin Terrier, Vladimir Tesar, Michelangelo Tesi, Augusto Vaglio, Dagmar Wandrei, Arthur White, Krzysztof Wójcik, Beyza Yaman, Mark A Little, Aladdin J Mohammad

**Affiliations:** 1 Clinical Sciences, Rheumatology, Lund University, Lund, Sweden; 2 School of Infection and Immunity, University of Glasgow, Glasgow, UK; 3 Department of Mathematical Science, University of Durham, Durham, UK; 4 Telemedicine Technologies, Liège, Belgium; 5 ADAPT SFI Centre, School of Computer Science and Statistics, Trinity College Dublin, Dublin, Ireland; 6 Department of Nephrology, General University Hospital, Prague, Czech Republic; 7 First Faculty of Medicine, Charles University, Prague, Czech Republic; 8 National Referral Center for Rare Systemic Autoimmune Diseases, Hospital Cochin, Paris, France; 9 Université Paris Cité, Paris, France; 10 Department of Rheumatology and Clinical Immunology, Universitat zu Lubeck, Lubeck, Germany; 11 Division of Molecular Biology and Clinical Genetics, Jagiellonian University Medical College, Krakow, Poland; 12 2nd Department of Internal Medicine, Jagiellonian University Medical College, Krakow, Poland; 13 French Vasculitis Study Group, Paris, France; 14 Trinity Kidney Centre, Trinity Translational Medicine Institute, Trinity College Dublin, Dublin, Ireland; 15 Department of Clinical Sciences, Lund University, Lund, Sweden; 16 Department of Endocrinology, Nephrology and Rheumatology, Skåne University Hospital, Lund, Sweden; 17 General University Hospital in Prague, Praha, Czech Republic; 18 Nephrology and Dialysis Unit, Meyer Children’s Hospital IRCCS, Firenze, Italy; 19 Clinical Trials Unit, Medical Center, University of Freiburg Faculty of Medicine, Freiburg, Germany; 20 School of Computer Science and Statistics, Trinity College Dublin, Dublin, Ireland; 21 Department of Medicine, University of Cambridge, Cambridge, UK

**Keywords:** systemic vasculitis, epidemiology, granulomatosis with polyangiitis, quality indicators, health care

## Abstract

**Objectives:**

This study aims to describe the data structure and harmonisation process, explore data quality and define characteristics, treatment, and outcomes of patients across six federated antineutrophil cytoplasmic antibody-associated vasculitis (AAV) registries.

**Methods:**

Through creation of the vasculitis-specific Findable, Accessible, Interoperable, Reusable, VASCulitis ontology, we harmonised the registries and enabled semantic interoperability. We assessed data quality across the domains of uniqueness, consistency, completeness and correctness. Aggregated data were retrieved using the semantic query language SPARQL Protocol and Resource Description Framework Query Language (SPARQL) and outcome rates were assessed through random effects meta-analysis.

**Results:**

A total of 5282 cases of AAV were identified. Uniqueness and data-type consistency were 100% across all assessed variables. Completeness and correctness varied from 49%–100% to 60%–100%, respectively. There were 2754 (52.1%) cases classified as granulomatosis with polyangiitis (GPA), 1580 (29.9%) as microscopic polyangiitis and 937 (17.7%) as eosinophilic GPA. The pattern of organ involvement included: lung in 3281 (65.1%), ear-nose-throat in 2860 (56.7%) and kidney in 2534 (50.2%). Intravenous cyclophosphamide was used as remission induction therapy in 982 (50.7%), rituximab in 505 (17.7%) and pulsed intravenous glucocorticoid use was highly variable (11%–91%). Overall mortality and incidence rates of end-stage kidney disease were 28.8 (95% CI 19.7 to 42.2) and 24.8 (95% CI 19.7 to 31.1) per 1000 patient-years, respectively.

**Conclusions:**

In the largest reported AAV cohort-study, we federated patient registries using semantic web technologies and highlighted concerns about data quality. The comparison of patient characteristics, treatment and outcomes was hampered by heterogeneous recruitment settings.

**WHAT IS ALREADY KNOWN ON THIS TOPIC**

- Semantic web technologies enable simultaneous privacy preserving querying of multiple distributed data sources.

**WHAT THIS STUDY ADDS**

- This is the first study to demonstrate successful implementation of federated data integration in a rheumatic disease.
- This is the first study to address issues in completeness and correctness of European vasculitis registry data.
- Comparison of patient characteristics, treatments and outcomes of antineutrophil cytoplasmic antibody-associated vasculitis (AAV) across European registries is presented in largest AAV cohort ever reported.

**HOW THIS STUDY MIGHT AFFECT RESEARCH, PRACTICE OR POLICY**

- This first attempt to federate AAV patient registries shows the potential for international benchmarking of patient care using distributed registries with variable registry design.
- This study highlights the need for structured assessment of data quality to allow for scientifically robust research and reliable decision-making.
- Data source federation is not limited to clinical registries, the infrastructure may be readily expanded to include genomic and postgenomic research data and the methodology is readily scalable to other rheumatic diseases.

## Introduction

Antineutrophil cytoplasmic antibody (ANCA)-associated vasculitis (AAV) is a group of systemic autoimmune disorders characterised by inflammation and destruction of predominantly small blood vessels. AAVs encompass three disease subtypes, sharing clinical and pathological features and association with ANCA; granulomatosis with polyangiitis (GPA), microscopic polyangiitis (MPA) and eosinophilic GPA (EGPA). All three types of AAV are rare, with a combined annual incidence rate of about 30 per million adults.^1^ A sufficiently large number of observations are required for reliable statistical inference of patient data, but low incidence and consequently small sample sizes of observational cohorts present a major barrier to clinical research in AAV.

A growing number of clinical trials in vasculitis, and the subsequent need to collect long-term data on treatment safety and efficacy in routine care, has led to development of several vasculitis registries across Europe.^2 3^ These registries play an increasing role in research, patient care and healthcare planning. In the FAIRVASC (Findable, Accessible, Interoperable, Reusable, VASCulitis) project, AAV data from six well-established European vasculitis registries are federated.^4^ The project has been developed in the context of the European Joint Programme on Rare Diseases (EJPRD), a Europe-wide initiative to overcome challenges related to data fragmentation in rare disease research.^5^ Using a semantic-web approach, the challenge of heterogeneous data structures and semantics have been addressed by the creation of a vasculitis-specific FAIRVASC ontology, as well as aligning the project with the FAIR principles of scientific data management, making data findable, accessible, interoperable and reusable.^6–8^

To allow valid and reliable decision-making based on scientific data, it is important to ensure data are of high quality. The literature proposes a diverse range of dimensions and methodologies to describe and measure data quality.^9–11^ However, the quality of European vasculitis registries or vasculitis research data has rarely been formally assessed.

Our aims in this observational retrospective cohort study are to (1) describe the data structure, federation and harmonisation process, (2) explore data quality and (3) give an overview on the baseline characteristics, treatment and outcomes of AAV patients across six European registries.

## Methods

### Registry federation

**Figure 1:**
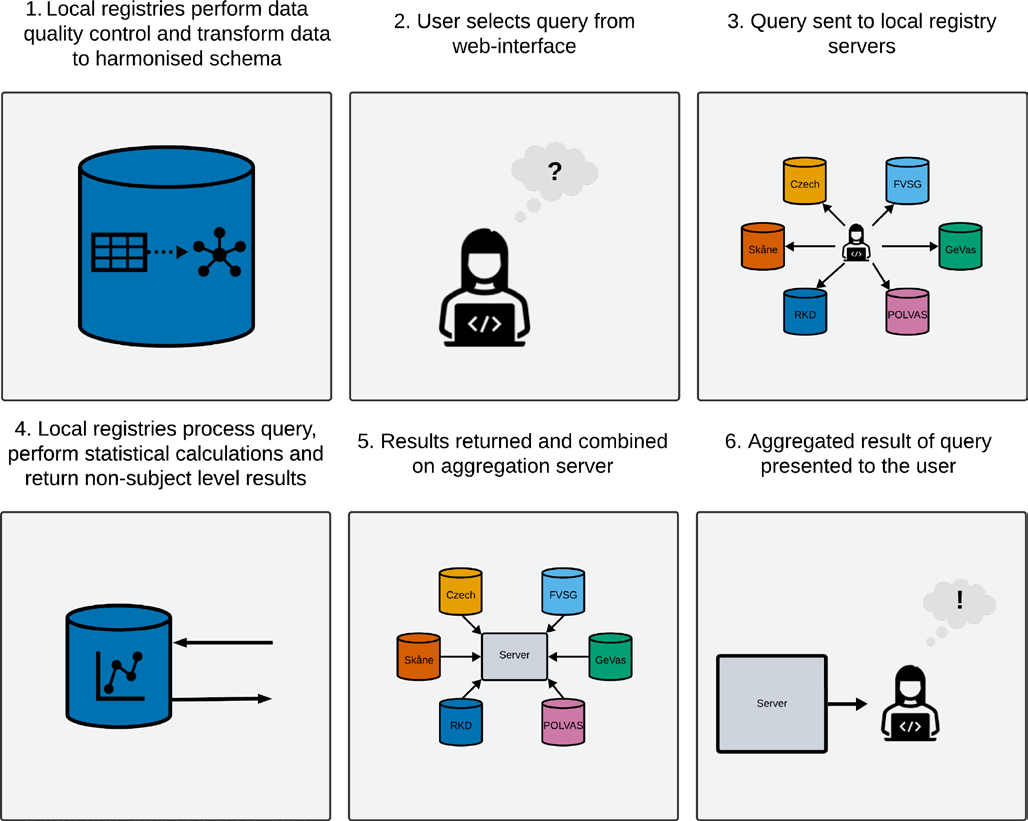
FAIRVASC project workflow. Registry data are quality controlled and harmonised to a common schema. Harmonised data are uploaded to a local server with an exposed endpoint. A researcher dispatches a predefined query from a web interface to the local servers. Statistical calculations are performed locally, and non-subject level results sent back. Data are combined and presented to the web-interface user. FAIRVASC, Findable, Accessible, Interoperable, Reusable, VASCulitis.

This descriptive study is part of a data reuse project aiming to federate multiple existing vasculitis registries. Federation stands in contrast to traditional centralisation where multiple local independent datasets are combined in a central data pool for analysis. Instead, data are held within the firewall of its home institution to be queried and analysed remotely, addressing data privacy, access and security concerns inherent in establishing a central data pool. Through federation local registries retain control over data access rights in compliance with their ethical approvals and/or informed consent of registry participants. In FAIRVASC, data federation is enabled through a web interface,^12^ following previous approaches for medical data linkage.^13 14^ Participant registries keep autonomous control over how registry data are represented internally, while exposing an endpoint to allow federated retrieval of non-subject level data through the website (figure 1).

### The registries

The participating registries are the Czech Registry of AAV,^15^ the French Vasculitis Study Group registry (FVSG),^16^ the Joint Vasculitis Registry in German-speaking Countries (GeVas),^17^ the Polish Vasculitis Registry (POLVAS),^18^ Ireland’s Rare Kidney Disease (RKD) registry^19^ and Sweden’s Skåne Vasculitis Cohort.^1^ These registries were selected through the registries working group of the European Vasculitis Society (EUVAS) as a combination of highly mature and recently established registries and reflecting a balance of rheumatology and nephrology-led initiatives. A key criterion was the availability of outcome data (eg, death and end-stage kidney disease (ESKD)), thereby excluding some important registries such as UKIVAS. The characteristics of the registries, including location and period of recruitment, tools for data collection and follow-up, are summarised in table 1.

**Table 1:** Meta-data summary of the FAIRVASC registries

	Czech*	FVSG	GeVas	POLVAS	RKD	Skåne
Current no of AAV cases	335	2804	169	936	668	374
Types of AAV*	EGPA, MPA, GPA, Unspecified AAV	EGPA, MPA, GPA	EGPA, MPA, GPA, Unspecified AAV	EGPA, MPA, GPA, Unspecified AAV	EGPA, MPA, GPA, Unspecified AAV	EGPA, MPA, GPA
Classification method	EMA algorithm, Clinical	CHCC 2012, ACR 1990	CHCC 2012, ACR 1990, Clinical	CHCC 2012, ACR 1990	EMA algorithm	EMA algorithm
Recruitment area	National	National	National	National	National	Regional
Name of recruitment area	Czech Republic	France	Germany	Poland	Ireland	Skåne, Sweden
Setting	Secondary/tertiary care	Secondary/tertiary care	Vasculitis centres	Secondary/tertiary care	Secondary/tertiary care	Population coverage
Dominant medical specialties	Nephrology	All	All	All	Nephrology	All
Study design	Mixed retrospective/prospective	Prospective cohort study	Mixed retrospective/prospective	Retrospective cohort study	Mixed retrospective/prospective	Mixed retrospective/prospective
Period of recruitment	2009–31 January 2023	1983–31 January 2023	2019–31 January 2023	2016–31 January 2023	2012–31 January 2023	1997–31 December 2019
Follow-up	Longitudinal	Longitudinal	Longitudinal	Longitudinal	Longitudinal	Longitudinal
Data source	Encounter based	Encounter based	Encounter based	Chart review	Encounter based	Chart review

*In this study, only single-centre data starting from 2013 is presented. EMA,^41^ CHCC 2012, 2012 Revised International Chapel Hill Consensus Conference Nomenclature of Vasculitides,^42^ ACR 1990 criteria.^43 44^

AAV, anti-neutrophil cytoplasmic antibody-associated vasculitis; ACR, American College of Rheumatology; EGPA, Eosinophilic granulomatosis with polyangiitis; EMA, European Medicines Agency; FAIRVASC, Findable, Accessible, Interoperable, Reusable, VASCulitis; FVSG, French Vasculitis Study Group; GeVas, Joint Vasculitis Registry in German-speaking Countries; GPA, Granulomatosis with polyangiitis; MPA, Microscopic polyangiitis; POLVAS, Polish Vasculitis Registry; RKD, Rare Kidney Disease.

We included all patients with a diagnosis of AAV, including GPA, MPA, EGPA and unspecified AAV, regardless of classification method. Two (Czech and RKD) of the registries excluded patients with a classified diagnosis of AAVs that were positive for anti-glomerular basement membrane antibodies. In four registries (FVSG, Skåne and POLVAS, RKD), unspecified AAVs were excluded or not recruited.

### Data harmonisation

The data structure and variables collected across the registries are different. To enable semantic interoperability, we harmonised the definitions of data elements and their corresponding values and converted registry data into a structure that adheres to a harmonised schema. This was achieved by the creation of the vasculitis-specific FAIRVASC ontology, that is, a representation, formal naming and definition of the categories, properties and relations between the concepts, data and entities within the registries.^6^ In the conversion to the harmonised schema, we uplifted heterogeneous registry data to a machine-readable format using the declarative mapping language ‘RDB to RDF Mapping Language’ and enriched data across the registries by adding semantic meaning to data using terms from standardised ontologies, such as the Orphanet Rare Disease Ontology.^20^ The use of ontologies is a key part of the FAIR principles of scientific data management and stewardship, an initiative putting emphasis on machine readability and reusability of data.^7 21^ The development of the FAIRVASC ontology and semantic integration of vasculitis data have previously been described in detail by McGlinn *et al*.^22^ This harmonisation process results in transformation of unstructured relational data to a knowledge graph data format.

### Data quality

We assessed data quality in the registries across four core domains: uniqueness, consistency, completeness and correctness. These domains were selected in collaboration with the European Institute for Innovation through Health Data (i~HD), based on a pool of nine candidate dimensions in the i~HD data quality assessment framework.^23^ In assessing uniqueness (absence of duplication of data), we investigated duplicate patient identifiers and potential cases of readmission of the same patient under another unique identifier within each registry. Consistency was assessed by examining nine key variables in AAV research (patient gender, date of birth, immunoassay ANCA-type, Birmingham Vasculitis Activity Score (BVAS) at diagnosis, serum creatinine at diagnosis, C reactive protein at diagnosis, induction treatment, date of death and date of ESKD) for the appropriate data type according to the registries’ data dictionaries. Logic tests were applied to variables containing dates (eg, ‘Was date of death greater than date of diagnosis’). Plausibility tests of numeric values were performed for two variables (eg, ‘Was serum creatinine at diagnosis within a biologically plausible range of 0–5000 µmol/L’). For the completeness domain, the amount of missing data was assessed across the same variables. Correctness of data entry of the variables described above was assessed in each registry against an electronic health record for at least ten patients per registry. Based on these four domains, we developed a data quality worksheet (online supplemental file 1). Using this worksheet, data quality was assessed at each registry site by local investigators. The results of the data quality assessment are presented as variable specific percentages, stratified by registry.

10.1136/ard-2023-224571.supp1 Supplementary data

### Patient characteristics retrieval

We analysed patients recruited to the FAIRVASC registries with a diagnosis of AAV up to 31 January 2023, with descriptive analysis of demographics, type of diagnosis, organ involvement, serology, serum creatinine level at diagnosis, treatments, follow-up and outcome. Continuous variables are summarised with a mean and SD. Categorical variables are summarised as frequencies. We estimated the incidence rate of ESKD and all-cause mortality rate combined across the registries per 1000 person-years with a 95% CI using random effects meta-analyses. The definition of ESKD differed slightly between the registries (online supplemental file 2). The aggregated patient characteristics were retrieved from the knowledge graphs of the registries through the semantic query language SPARQL (online supplemental file 1), and random effects meta-analysis was performed using the R package *metafor*.^24^ As the federated queries did not allow for the flow of subject level data from one site to another or to a centralised server, the investigation of summary statistics requiring the global rank of data to be known (eg, quantiles) could not be assessed.

### Patient and public involvement

The sharing of data in this study concerns aggregated patient data and is thus not governed by general data protection regulations. As health data are sensitive and rare disease data difficult to fully anonymise, we, in this study, chose to federate aggregated registry data, as opposed to develop and transfer data to a centralised data pool. This allows for sustainability through real-time updates of data presented, rigorous data access control by the data owner and scalability to include additional mature and emerging vasculitis registries. Patients and patient advocacy organisations have been involved in the design and conduct of the FAIRVASC project from inception. This ensures consideration of patient priorities and perspectives regarding the ethics, privacy and confidentiality of the federated research approach, the design of this study and future communication and dissemination of this work.

## Results

### Patient characteristics and treatment

A total of 5282 patients (2568 (48.6%) women) were included across the 6 registries. Of these, 2754 (52.1%) were classified as GPA, 1580 (29.9%) MPA and 937 (17.7%) as EGPA according to different sets of international classification criteria or clinical diagnosis. The mean age at diagnosis was 56.0±16.7 years. There were 1840 (51.2%) PR3-ANCA positive, 1506 (41.8%) MPO-ANCA positive and 219 (9.0%) immunoassay ANCA negative patients. Pulmonary involvement was reported in 3281 (65.1%), ear-nose-throat involvement in 2860 (56.7%) and kidney involvement in 2534 (50.2%). Cardiovascular and abdominal involvement were present in 822 (16.3%) and 658 (13.4%), respectively. The mean creatinine level at diagnosis was 198±266 µmol/L across the registries (table 2).

**Table 2:** Patient characteristics summary stratified by registry

	Czech	FVSG	GeVas	POLVAS	RKD	Skåne	Total
Total n of patients, n (%)	335 (100)	2804 (100)	169 (100)	932 (100)	668 (100)	374 (100)	5282 (100)
Demography							
Age at diagnosis, mean (SD)*	60.1 (15.2)	55.1 (16.5)	59.5 (15.3)	50.5 (15.9)	59.1 (15.6)	64.9 (16.2)	56.0 (16.7)
Men, n (%)	169 (50.4)	1441 (51.4)	83 (49.1)	431 (46.2)	384 (57.5)	200 (53.5)	2708 (51.3)
Women, n (%)	166 (49.6)	1357 (48.4)	86 (50.9)	501 (53.8)	284 (42.5)	174 (46.5)	2568 (48.6)
Diagnosis							
GPA, n (%)	143 (42.7)	1390 (49.6)	85 (50.3)	645 (69.3)	299 (44.7)	192 (51.3)	2754 (52.1)
MPA. n (%)	178 (53.1)	683 (24.4)	54 (31.9)	169 (18.2)	337 (50.4)	159 (42.5)	1580 (29.9)
EGPA, n (%)	5 (1.5)	731 (26.1)	28 (16.6)	118 (12.7)	32 (4.8)	23 (6.1)	937 (17.7)
Unspecified AAV, n (%)	8 (2.4)	†	2 (1.2)	‡	‡	†	10 (0.2)
ANCA§							
PR3-ANCA positive, n (%)	147 (44.1)	650 (50.7)	79 (46.8)	457 (55.9)	320 (47.9)	187 (50.0)	1840 (51.2)
MPO-ANCA positive, n (%)	170 (51.1)	615 (47.9)	61 (36.1)	173 (21.1)	326 (48.8)	161 (43.0)	1506 (41.8)
ELISA negative, n (%)	16 (4.8)	¶	26 (15.4)	134 (16.4)	17 (2.5)	26 (6.9)	219 (9.0)
S-creatinine at diagnosis, mean (SD)**	222 (149)††	171 (282)	156 (187)	¶	288 (263)	224 (223)	198 (266)
Organ pattern involvement ‡‡							
General, n (%)	208 (62.7)	2093 (81.3)	155 (92.3)	823 (88.6)	258 (38.6)	277 (74.5)	3814 (75.6)
Mucocutaneous/eye, n (%)	37 (11.1)	1359 (52.8)	67 (39.9)	434 (46.7)	204 (30.5)	66 (17.7)	2167 (43.0)
Ear-nose-throat, n (%)	112 (33.7)	1595 (61.9)	94 (55.9)	622 (67.0)	281 (42.1)	156 (41.2)	2860 (56.7)
Lung, n (%)	160 (48.2)	1792 (69.6)	112 (66.7)	682 (73.4)	338 (50.1)	197 (52.9)	3281 (65.1)
Cardiovascular, n (%)	7 (2.1)	631 (24.5)	17 (10.1)	127 (13.7)	20 (2.9)	20 (5.4)	822 (16.3)
Abdominal, n (%)	10 (3.0)	480 (18.6)	11 (6.6)	114 (12.3)	34 (5.1)	9 (2.4)	658 (13.4)
Kidney, n (%)	310 (93.4)	720 (27.9)	108 (64.3)	575 (61.9)	564 (84.4)	257 (69.1)	2534 (50.2)
Nervous, n (%)	51 (15.4)	1209 (46.9)	64 (38.1)	266 (28.6)	96 (14.3)	51 (13.7)	1737 (34.4)
Induction treatment§§							
Oral cyclophosphamide, n (%)	¶¶	7 (0.9)	0 (0)	***	222 (33.6)	128 (35.1)	357 (18.4)
IV cyclophosphamide, n (%)	¶¶	389 (51.1)	87 (58.4)	734 (79.6)***	340 (51.4)	166 (45.5)	1716 (60.0)
Rituximab, n (%)	¶¶	112 (14.7)	75 (50.3)	112 (12.1)	177 (26.7)	29 (7.9)	505 (17.7)
Plasma exchange, n (%)	88 (31.1)	44 (5.8)	9 (6.0)	81 (8.9)	137 (20.7)	62 (16.9)	421 (13.4)
Intravenous glucocorticoids, n (%)	258 (91.2)	248 (32.6)	122 (81.9)	699 (75.8)	367 (55.5)	41 (11.2)	1735 (55.2)
Maintenance treatment†††	¶¶	¶¶					
Azathioprine, n (%)			26 (37.7)	357 (41.7)	322 (57.2)	206 (62.2)	911 (50.1)
Rituximab, n (%)			44 (63.8)	19 (2.2)	163 (28.9)	56 (16.9)	282 (15.5)
Methotrexate, n (%)			0 (0)	210 (24.5)	0 (0)	60 (18.1)	270 (14.8)
Mycophenolate mofetil, n (%)			0 (0)	172 (20.1)	122 (21.7)	51 (15.4)	345 (18.9)
Outcome							
Death, n (%)	56 (16.7)	350 (12.5)	3 (1.8)	113 (12.4)	127 (19.0)	187 (50.0)	836 (15.8)
ESKD, n (%)‡‡‡	49 (24.1)§§§	298 (10.6)	5 (2.9)	145 (15.9)	127 (19.0)	55 (14.7)	679 (13.2)
Follow-up time in years, mean (SD)	3.4 (3.3)	6.4 (5.7)	0.8 (0.9)	6.1 (5.8)	7.3 (6.8)	7.9 (6.2)	6.2 (5.8)

If not commented complete data. All percentages presented as valid percentages.

*Available for n: 334 (Czech), 2529 (FVSG), 169 (GeVas), 901 (POLVAS), 666 (RKD), 374 (Skåne), 4973 (Total).

†Not included in registry collection.

‡Excluded in registry harmonisation.

§Available for n: 333 (Czech), 1283 (FVSG), 169 (GeVas), 774 (POLVAS), 668 (RKD), 374 (Skåne), 3601 (Total).

¶Not available information in registry design.

**Available for n: 253 (Czech), 2017 (FVSG), 138 (GeVas), 509 (RKD), 371 (Skåne), 3288 (Total).

††Cases on acute or chronic dialysis actively excluded

‡‡Available for n: 332 (Czech), 2574 (FVSG), 168 (GeVas), 929 (POLVAS), 668 (RKD), 372 (Skåne), 5043 (Total).

§§Available for n: 283 (Czech), 761 (FVSG), 149 (GeVas), 922 (POLVAS), 661 (RKD), 365 (Skåne), 3141 (Total).

¶¶Information not available due to registry design.

***Cannot differ administration route of cyclophosphamide in registry design. Presented is all cyclophosphamide.

†††Available for n: 69 (GeVas), 857 (POLVAS), 563 (RKD), 331 (Skåne), 1820 (Total).

‡‡‡End-stage kidney disease definitions: Czech (dialysis for >90 days; sustained CKD 5 (estimated glomerular filtration rate (eGFR)<15 mL/min/1.73 m^2^) for >90 days; and/or kidney transplantation), FSVG (dialysis for more than 30 days or death within 30 days of start of dialysis), GeVas (renal replacement therapy; sustained dialysis or CKD 5 in two succeeding visits), Skåne/POLVAS (sustained dialysis), RKD (dialysis for >90 days; sustained CKD 5 (eGFR<15 mL/min/1.73 m^2^) for >90 days; and/or kidney transplantation).

§§§Available for n 203.

AAV, antineutrophil cytoplasmic antibody associated vasculitis; EGPA, eosinophilic granulomatosis with polyangiitis; ESKD, end stage kidney disease; FVSG, French Vasculitis Study Group; GeVas, Joint Vasculitis Registry in German-speaking Countries; GPA, granulomatosis with polyangiitis; MPA, microscopic polyangiitis; POLVAS, Polish Vasculitis Registry; RKD, rare kidney disease.

### Data quality

**Figure 2:**
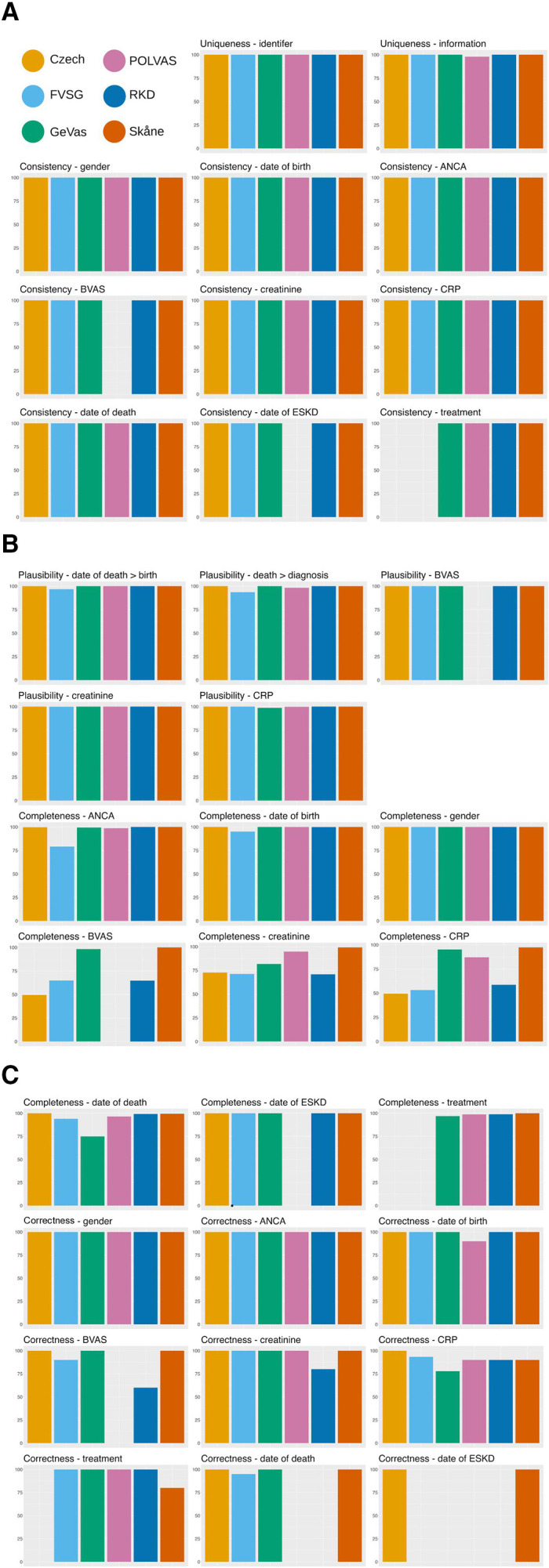
Data quality metrics per registry as percentages per key registry variables. (A) Data uniqueness (the absence of duplication of data) and data-type consistency. (B) Plausibility of data values and data completeness (the absence of missing data). (C) Data completeness (the absence of missing data) and correctness (assessed against available electronic health records). Variables that were missing from a registry because they were not targeted for collection are shown as the absence of a bar. Full data available in online supplemental table 4. ANCA, antineutrophil cytoplasmic antibody; BVAS, Birmingham Vasculitis Activity Score; CRP, C reactive protein; ESKD, end-stage kidney disease; FVSG, French Vasculitis Study Group; GeVas, Joint Vasculitis Registry in German-speaking Countries; POLVAS, Polish Vasculitis Registry; RKD, Rare Kidney Disease.

No duplicate patient identifiers were identified in the registries, although, in one registry, there were 2.2% potential duplicate entries (ie, whereby the same patient was entered under more than one unique identifier). Consistency of data type was 100% across all assessed variables present in the registries. Consistency for logic tests of dates was between 93.6% and 100% and between 98.7% and 100% for plausibility tests for numeric variables. Completeness for gender and date of birth ranged from 95.1% to 100% across all registries. Not all variables were present in all registries due to differences in registry design. Completeness for laboratory tests at diagnosis ranged from 49.5% to 99.2%. A BVAS assessment was available at diagnosis for 49.5%–100% of patients. In the registries which record induction treatment, at least one treatment type was recorded in 96.9% to 100% of the cases. In patients who died or reached ESKD by the end of follow-up, a date of death or date of ESKD was available in 75%–100%. Data correctness was 60.0%–100% across all variables and registries (figure 2A–C and online supplemental file 1).

### Treatment

Some information on induction treatment was collected in all registries, but the granularity of information differed. Cyclophosphamide could be assessed in five of six registries, and oral and intravenous use could be separated in four registries. Information about rituximab was present in five of six registries. The use of pulsed intravenous glucocorticoids and plasma exchange could be assessed in all registries. Oral and intravenous cyclophosphamide was used in 357 (18.4%) and in 1716 (60.0%), respectively, and rituximab was used in 505 (17.7%) patients. Plasma exchange was used in 421 (13.4%) and pulsed intravenous glucocorticoids in 1735 (55.2%). Maintenance treatment was recorded in 4 of 6 registries, with azathioprine used in 911 (50.1%) and rituximab in 282 (15.5%) (table 2).

### Outcome analysis

**Figure 3:**
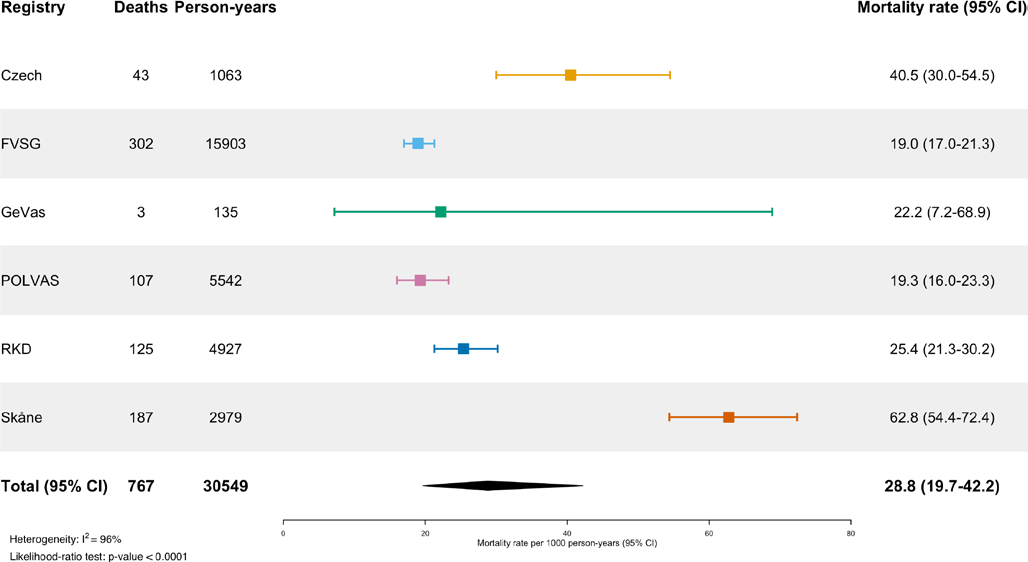
All-cause mortality rate per 1000 patient years. Forest plot showing estimated all-cause mortality rate per 1000 patient years. Per registry and pooled estimates shown with 95% CI. FVSG, French Vasculitis Study Group; GeVas, Joint Vasculitis Registry in German-speaking Countries; POLVAS, Polish Vasculitis Registry; RKD, Rare Kidney Disease.

**Figure 4:**
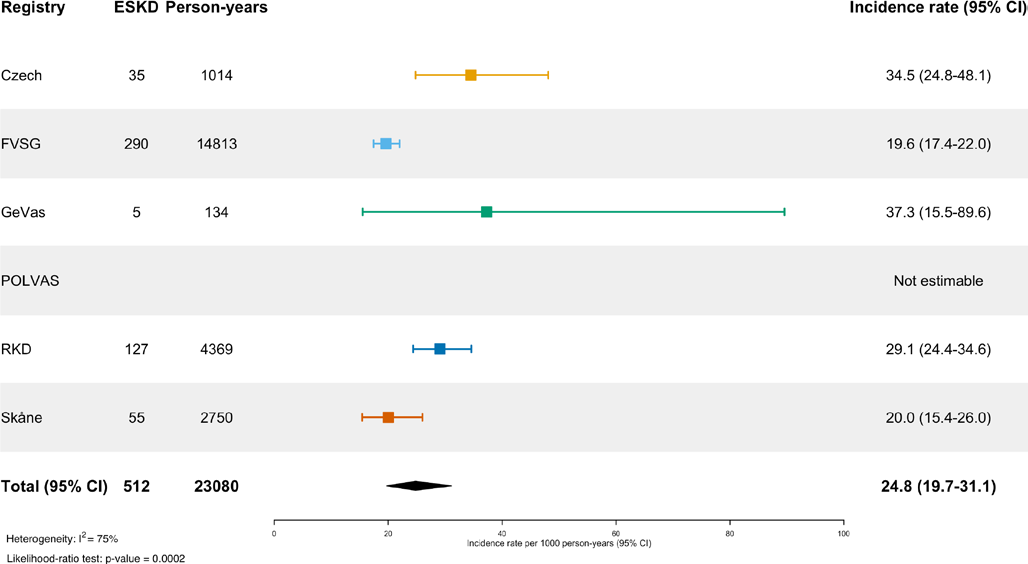
Incidence rate of end-stage kidney disease per 1000 patient years per registry. Forest plot showing estimated incidence rate of end-stage kidney disease per 1000 patient years. Per registry and pooled estimates shown with 95% CI. FVSG, French Vasculitis Study Group; GeVas, Joint Vasculitis Registry in German-speaking Countries; POLVAS, Polish Vasculitis Registry; RKD, Rare Kidney Disease.

The mean follow-up time was 6.2±5.8 years with a total of 30 548 person-years of follow-up. During follow-up, there were 767 deaths occurring with a known date of death, yielding a pooled all-cause mortality rate of 28.8 (95% CI 19.7 to 42.2) per 1000 patient-years (figure 3). The highest mortality rates were seen in the Skåne and Czech registries, 62.8 (95% CI 54.4 to 72.4) and 40.5 (95% CI 30.0 to 54.5), respectively. The pooled incidence-rate of ESKD was 24.8 (95% CI 19.7 to 31.1) per 1000 patient years (figure 4). There was considerable heterogeneity among the registries (I^2^=96% and I^2^=75%, respectively). The I^2^ statistic describes the percentage of variation across studies that is due to heterogeneity rather than chance.

## Discussion

In this observational retrospective cohort study, the largest yet reported, we describe the data structure and harmonisation process, explore data quality and give an overview on baseline characteristics and outcomes of AAV patients across six European patient registries within the FAIRVASC project.

Despite collaborative efforts in recent years, research data in vasculitis have remained fragmented, siloed, and is rarely standardised from the perspective of data interoperability. To allow federated analytics across multiple registries, a process of data harmonisation across data sources is needed.^14 25^ The FAIRVASC ontology provides a framework for harmonisation that is scalable to existing or emerging AAV registries and cohorts.^22^ The use of semantic web technologies in rare disease research data integration has precedents in the medical literature. Importantly, the RD-Connect project and EJPRD Virtual Platform are high-level data integration projects aiming to achieve interoperability over a wide range of rare diseases and data resources.^26 27^ Our development of a disease-specific ontology provides a framework suitable for clinical research. The cohort presented in this study is already of unprecedented size in AAV and will soon be extended by the addition of further AAV registries using the framework provided.

High-quality data are essential to allow reliable decision-making. Despite this, there are no formalised frameworks for assessment of data quality specific for rare disease research and no agreed thresholds recognised for what constitutes sufficient data quality. We assessed data quality based on the i~HD data quality assessment framework.^23^ Arguably, the critical threshold for data quality varies depending on what data will be used for. This study demonstrated high data quality regarding uniqueness and consistency across participating registries. However, laboratory values and disease activity assessments at diagnosis lacked in completeness. A correctness assessment, comparing a subset of registry data with that of health records, showed some non-matching information in all but one registry.

This highlights evident data quality issues in present day vasculitis registries. Lack of completeness and some missing data are an inevitable part of any real-life dataset and can be addressed statistically. Additionally, the completeness assessment does not investigate why data were missing and may, in some cases, reflect registry design rather than completeness. For example, a creatinine value at baseline may appear to be missing but would reasonably not be collected in a patient on acute dialysis. Clinical data collection is inherently subjective which may be reflected in the completeness assessment. BVAS has previously shown high interobserver reliability by Suppiah *et al*, but the study was limited by small numbers.^28^ Selecting an appropriate laboratory test value in relation to a date of diagnosis may also be subject to interpretation. Potential steps to improve data quality in vasculitis registries include further enhancement of data validation systems, automated input from electronic health records and targeted data collection to improve completeness and correctness of key variables.^29^

This cohort of 5282 AAV patients is the largest cohort of AAV ever reported. GPA was the most common diagnosis across the registries, while MPA and EGPA followed. The varying classification criteria used for registry inclusion and the wide temporal spread in the period of recruitment needs to be considered when interpreting these results. The low frequency of MPA in the FVSG registry is expected with registry inclusion starting in 1983 from a multidisciplinary perspective. MPA was recognised as a distinct disease subtype in the 1994 Chapel Hill Consensus Criteria.^30^ The high frequency of MPA in the Czech and RKD registries may be attributed to the nephrology-centred patient inclusion of these registries. The mean age at diagnosis in the Skåne registry was significantly higher than in the other registries. This can likely be explained by the Skåne registry’s population coverage design. This may indicate that, as in clinical trials, the oldest patient groups are underrepresented in patient registries.^31^ PR3 positivity was the most common ANCA pattern overall, largely following the frequency of diagnosis subtype, with MPO positivity being more common in registries recruiting primarily from a nephrology setting. We saw a large variance in organ pattern distribution of active disease across the registries, likely reflecting the different recruitment settings. These differences in disease phenotype need to be considered when assessing regional observational studies.

Intravenous cyclophosphamide was the most administered remission induction treatment, followed by oral cyclophosphamide and rituximab. The use of remission maintenance treatment could be assessed in four registries with azathioprine being most used, followed by rituximab and methotrexate. A comparison of the registries with regard to treatments used is hampered by the variability of disease severity of included cases, the differences in recruitment period and settings and the lack of data completeness in this domain. However, rituximab was more frequently used for both remission induction and maintenance in GeVas, the most recently developed registry, indicating a shift towards an increased use following the RITUXVAS, RAVE and MAINRITSAN trials.^32–34^

We saw small differences in the use of plasma exchange across the registries. These differences may lie in heterogeneity of recruitment settings, time of patient inclusion and the following patient sample, but data are lacking to support further discussion. However, the large differences in use of pulsed intravenous glucocorticoids across registries may reflect, at least in part, differences in clinical practice. In the population coverage Skåne registry, 11% of the patients received pulsed intravenous glucocorticoids, compared with 91% in the Czech, 82% in GeVas, 76% in POLVAS, 56% in RKD and 33% in the FVSG registry. Pulsed intravenous glucocorticoids may not confer clinical benefit while being associated with higher incidence of infections and diabetes.^35^ However, clinical trials are lacking, which reinforces the need for real-world data interpretation through such a combined analysis. The recently updated European Alliance of Associations for Rheumatology management guidelines highlight the limited evidence base for the use of pulsed intravenous glucocorticoids in remission induction of AAV and recommend the use only in life-threatening or organ-threatening disease.^36^ Our study indicates that use is highly variable across the registries. While the updated management guidelines may reduce differences in clinical practice across regions, further studies are needed to assess the benefit and risk of routine use in remission induction of AAV.

Under-reporting of outcomes is a potential issue in the observational prospective design of the source registries. There is considerable heterogeneity in the mortality rates across the registries, likely reflecting the different recruitment settings and follow-up control of the registries rather than differences in care, further strengthening the case that patient registries may underreport some patient groups. The higher mortality rate in the Skåne registry is explained by complete mortality data ascertainment through linkage with the Swedish National Board of Health and Welfare. The overall mortality rate in our study (28.8 per 1000 person-years) is also low compared with what is described in other cohorts.^37 38^ A similar heterogeneity in the rate of ESKD is seen. Here comparison is hampered by the different definitions of ESKD used and differences in recruitment. The analysis has not been limited to subjects with kidney involvement, the frequency of which differs greatly between the registries. Likewise, a comparison of the rate of ESKD with the literature is difficult due to variability in kidney outcome measures, statistical methods used and the characteristics of the cohorts studied. In this study 13% of the patients reached ESKD during follow-up with and incidence rate of 24.8 per 1000 person-years, comparable with the percentage (~18%) reaching ESKD in the long-term follow-up of EUVAS trials.^2^ When assessing outcome over registry data it is of importance to address the survival bias inherent in patient recruitment. Analysis performed in relation to the time of diagnosis presents an immortal time bias in the prevalent cases that may have been recruited to the registry years after disease onset. Likewise, only patients that survived until the time of recruitment are included. In this descriptive study, no attempt is taken to address this issue. Further analysis of mortality and kidney survival in AAV using this large cohort are planned.

Previous large-scale cohort studies in AAV have largely used clinical trial data, despite the well-documented problems of generalisability.^39^ With no specific exclusion criteria, the full spectrum of AAV was included in this study, with the registries recruiting patients through clinics across Europe, mostly from nephrology, rheumatology and internal medicine centres. While this might inhibit epidemiological comparisons across the registries, it more accurately reflects the full spectrum of disease than any single registry effort. The harmonisation approach is scalable and can readily include existing and emerging AAV registries. However, there are study limitations. Research across registries, with the associated varying data structures and data items collected, means that trade-offs are needed between generic data covering all registries and more specific data that may cover only some of the registries. To support the federation of a large European AAV dataset, the harmonised schema created is currently only covering key variables in observational AAV research. Thus, important outcome measures with limited availability (eg, patient-reported outcomes, disease damage, relapsing disease and cumulative glucocorticoid exposure) are not presented. Furthermore, the lack of standards for definitions to be used, and the variables to be collected in the registries, prevent direct comparison of, for example, ESKD or treatment choices. We cannot be certain that the indicated treatment in this study is the first line treatment, and the design of some registries does not allow the separation of induction and maintenance treatment use of rituximab and the separation of oral and intravenous cyclophosphamide. In addition, lack of granularity may affect the interpretation of baseline serum creatinine as we do not have data on acute dialysis at the time of diagnosis in most registries.

We aim to further use this large cohort to identify new data-driven phenotypic descriptors and create clinical risk models to support the prediction of prognosis. The interoperability allows for the potential future implementation of federated learning to enable joint modelling on data from all included registries. Seeking to address data governance and privacy concerns, federated learning approaches are, therefore, being developed to fit statistical models and algorithms (eg, the extended analysis of patient outcome, including adjusted proportional hazards models) to distributed datasets without exchanging the data itself.^40^ This is key in projects where data privacy concerns restrict data access and prohibits transfer of data to a centralised pool. A successful implementation of federated learning holds the potential for precision medicine on a large-scale while respecting privacy concerns, overcoming the limitations of approaches that require a single pool of centralised data.

The harmonisation schema developed within this project is readily extendable to existing and emerging AAV registries. This is simplified through the development of the EUVAS ‘Model registry’, providing a framework for the prospective collection of a defined set of data items.^3^ Working in conjunction with the FAIRVASC harmonisation schema, this is building the foundation for further data integration and interoperability of AAV registries. Data source federation is not limited to clinical registries and the infrastructure may be expanded to include genomic and postgenomic research data. The methodology is also scalable to other rheumatic diseases where research suffers from small sample sizes and governance hurdles of international data sharing.

In this observational retrospective cohort study, we present data from six well-established European AAV registries represented in knowledge graphs, supporting scalable federated benchmarking of care. A data quality assessment indicated high levels of data quality regarding uniqueness and consistency of data, but there are data quality concerns regarding completeness and correctness for some of the key variables in AAV research. Our analysis of patient characteristics and outcomes of AAV across Europe is difficult to interpret due to differences in registry patient inclusion setting and period of recruitment. However, there is variability in patient care not adequately explained by these differences. This first attempt to federate AAV patient registries demonstrates the potential for benchmarking of patient care across Europe and the development of precision medicine at large-scale while respecting patient privacy concerns using a federated approach.

## Data Availability

Data may be obtained from a third party and are not publicly available. All data relevant to the study are included in the article or uploaded as supplementary information.
