# Supplementary material for: Data quality and patient characteristics in European ANCA-associated vasculitis registries: data retrieval by federated querying
Source: Ann Rheum Dis. 2023 Oct 31;83(1):112–20. doi: 10.1136/ard-2023-224571 (PMC10804071; doi:10.1136/ard-2023-224571)
Supplement: Supplementary data [file ard-2023-224571supp001.pdf]

Supplemental 1

Data quality worksheet

Thanks for working through these steps for your registry. Please document your output in CSV format, as per the accompanying example spreadsheet, then copy to this master sheet. Sheet 3 in the spreadsheet is a 'mini-data dictionary' which should help you match up the tasks below to the correct output variable name. Also please document the process as you go, for example by describing each data analysis step in a simple Word document or in an annotated data analysis script (e.g. an R or Python script with lots of comments). Don't get stuck on any one task – it may be that some tasks don't suit the data structure of your registry. Please get in touch with me if any one step is proving tricky.

Best Wishes,
Michelangelo

Data preparation:

- When reading in/cleaning data, ensure missing data will be recognised as such (e.g. some datasets represent missing data with a "." [full stop] or a numeric code) ● Please include ANCA-associated vasculitis patients only in your analysis. These include patients with granulomatosis with polyangiitis (GPA, formerly Wegener's), microscopic polyangiitis (MPA) and eosinophilic granulomatosis with polyangiitis (EGPA, formerly Churg-Strauss). Other AAV types might include "renal limited vasculitis" and "AAV unclassified". Examples of non-AAV vasculitis to exclude are GCA, Takayasu, Kawasaki and IgA vasculitis.

Diagnosis stratification:
With the present round of the data quality (DQ) assessment, we aim at stratifying the output based on the diagnosis. Namely, the goal is to perform the whole analysis (detailed below) for each of the following cohorts:

- All AAV patients
- EGPA patients
- GPA patients
- MPA patients

1. First document the date the data for DQ analysis was extracted

2. Uniqueness
    a. First report the total number of patient IDs (including duplicates). If the registry is encounter (or visit) based, also report the total number of visits (including duplicates). These will act as denominators.
    b. Report the number of duplicate entries for any patient identifier codes, as a raw number.
    c. If the registry is encounter / visit based – report the number of duplicate encounters, as a raw number.

d. Report the number of patients who have been entered more than once with separate IDs. First identify possible cases by finding individuals who share both the same date of birth and gender. Then further compare these individuals either by hand or using other variables (such as approximate date of diagnosis, date of death) to determine if a duplicate was entered. (Don't remove duplicates yet, this will be part of the next stage of data quality improvement).

3. Consistency
   a. For each of the following 'core DQ' variables, please count the number of cases where the variable of interest is in the correct data type (e.g. characters, binary, numeric, integer, date). We presume there should be one value per patient for these variables.
      i. Gender
      ii. Date of birth (or year of birth)
      iii. ANCA specificity
      iv. Presence of a one specific comorbidity at diagnosis (e.g. check diabetes if present in your registry)
      v. BVAS score / organ involvement at diagnosis
      vi. Creatinine at diagnosis
      vii. CRP at diagnosis
      viii. Induction Treatment
      ix. Date of death
      x. Date of end stage kidney disease (ESKD)
   b. Plausibility tests: return the number of cases for which the following statements are "true" (for 3b.i please return also the N°of cases with available Date of Birth and Date of Death data, and similarly for 3b.ii too)
      i. Is date of death >= date of birth (if patient deceased)
      ii. Is date of death >= date of diagnosis (if patient deceased)
      iii. Is BVAS (at diagnosis) within the plausible range? (0-63)
      iv. Is creatinine (at diagnosis) within a plausible range? (e.g. 0 – 5000 micromol/L)
      v. Is CRP (at diagnosis) within a plausible range? (e.g. 0 - 1000 mg/L)

4. Completeness
   a. Quantify missing data for the first eight core DQ variables. Please report as number of complete cases (i.e. number of cases with available data for the variable of interest).

      Please note: it might be the case that, in your registry, the absence of induction treatment data reflects the lack of any induction treatment (in the clinical history of the patient) rather than an actual missingness due to the data not entered. This might require a check of the clinical record.
   b. Quantify missing data for the last two of the core DQ variables as follows:
      i. How many 'dates of death' values missing amongst deceased patients (and also report the number of deceased patients)
      ii. How many 'ESKD date' values missing amongst ESKD patients (and also report the number of ESKD patients)

5. Correctness - This is a measure of how much the entered data adheres to its source. Check whether the core DQ variables are correct for 10 real patients against a 'gold standard' source e.g. clinical record, report as a percentage for each variable

BMJ Publishing Group Limited (BMJ) disclaims all liability and responsibility arising from any reliance placed on this supplemental material which has been supplied by the author(s)

Specification on BVAS, creatinine and CRP at diagnosis: these variables are considered as values at diagnosis only if they were measured within a two week timespan from the diagnosis date (i.e. within two weeks before diagnosis or two weeks after diagnosis).

Supplemental table 2

Table 2. Registry definitions of end-stage kidney disease

Registry	Definition of end-stage kidney disease
Czech	Dialysis for >90 days; sustained CKD 5 for >90 days; and/or kidney transplantation
FVSG	Dialysis for more than 30 days or death within 30 days of start of dialysis
GeVas	Renal replacement therapy; sustained dialysis or CKD 5 in two succeeding visits
POLVAS	Sustained dialysis
RKD	Dialysis for >90 days; sustained CKD 5 for >90 days; and/or kidney transplantation
Skåne	Sustained dialysis

CKD 5: chronic kidney disease stage 5 (eGFR < 15 ml/min/1.73m^2)

Supplemental 3

```
# Query 1: Total N of the registry

PREFIX fvc: <http://w3id.org/FAIRVASC#>
PREFIX bvas: <http://w3id.org/BVAS#>

SELECT (COUNT(DISTINCT ?patient) AS ?total_n)

WHERE{

?patient a fvc:Patient .
}

# Query 2a: Mean age in registry

PREFIX fvc: <http://w3id.org/FAIRVASC#>
PREFIX bvas: <http://w3id.org/BVAS#>

SELECT
(AVG(?ageatdiagnosis) AS ?avg_age)

WHERE {
?patient a fvc:Patient.
?patient fvc:hasPatientOverview ?PatientOverview.
?PatientOverview fvc:hasDiagnosis ?Diagnosis.
?Diagnosis fvc:ageAtDiagnosis ?ageatdiagnosis.

}

# Query 2b: Number of patients with available age information in registry
```

```
PREFIX fvc: <http://w3id.org/FAIRVASC#>
PREFIX bvas: <http://w3id.org/BVAS#>

SELECT
(COUNT(DISTINCT ?patient) AS ?n_available_age)

WHERE {
?patient a fvc:Patient.
?patient fvc:hasPatientOverview ?PatientOverview.
?PatientOverview fvc:hasDiagnosis ?Diagnosis.
?Diagnosis fvc:ageAtDiagnosis ?ageatdiagnosis.

}

# Query 2c: Age variance in registry (this has to be sqrt to retrieve standard deviation of age
which can't be done in pure SPARQL)

PREFIX fvc: <http://w3id.org/FAIRVASC#>
PREFIX bvas: <http://w3id.org/BVAS#>


SELECT(SUM((?ageatdiagnosis-?avg_age)*(?ageatdiagnosis-?avg_age))/(COUNT(?patient) -
1) as ?variance)

WHERE {
?patient a fvc:Patient.
?patient fvc:hasPatientOverview ?PatientOverview.
?PatientOverview fvc:hasDiagnosis ?Diagnosis.
?Diagnosis fvc:ageAtDiagnosis ?ageatdiagnosis.
 {
SELECT
(AVG(?ageatdiagnosis) AS ?avg_age)
WHERE {
?patient a fvc:Patient.
?patient fvc:hasPatientOverview ?PatientOverview.
?PatientOverview fvc:hasDiagnosis ?Diagnosis.
?Diagnosis fvc:ageAtDiagnosis ?ageatdiagnosis.
}
  }
}

# Query 2d: Sum of squared registry deviations from global mean age

PREFIX fvc: <http://w3id.org/FAIRVASC#>
PREFIX bvas: <http://w3id.org/BVAS#>
```

```
SELECT(SUM((?ageatdiagnosis-56.0)*(?ageatdiagnosis-56.0)) as ?reg_sumsquare)

WHERE {
?patient a fvc:Patient.
?patient fvc:hasPatientOverview ?PatientOverview.
?PatientOverview fvc:hasDiagnosis ?Diagnosis.
?Diagnosis fvc:ageAtDiagnosis ?ageatdiagnosis.
}


# Query 3a: Number of women in the registry

PREFIX fvc: <http://w3id.org/FAIRVASC#>
PREFIX bvas: <http://w3id.org/BVAS#>

SELECT (COUNT(DISTINCT ?patient) AS ?female_n)

WHERE {
?patient a fvc:Patient.
?patient fvc:gender ?gender.
FILTER (?gender =
"http://identifiers.org/ncit:C16576"^^<http://www.w3.org/2001/XMLSchema#anyURI>)
}

# Query 3b: Number of men in the registry

PREFIX fvc: <http://w3id.org/FAIRVASC#>
PREFIX bvas: <http://w3id.org/BVAS#>

SELECT (COUNT(DISTINCT ?patient) AS ?male_n)

WHERE {
?patient a fvc:Patient.
?patient fvc:gender ?gender.
FILTER (?gender =
"http://identifiers.org/ncit:C20197"^^<http://www.w3.org/2001/XMLSchema#anyURI>)
}

# Query 3c: Total number of patients where gender information is available

PREFIX fvc: <http://w3id.org/FAIRVASC#>
PREFIX bvas: <http://w3id.org/BVAS#>

SELECT (COUNT(DISTINCT ?patient) AS ?n_gender)

WHERE {
?patient a fvc:Patient.
```

```
?patient fvc:gender ?gender.

}

# Query 4a: Number of GPA patients in the registry

PREFIX fvc: <http://w3id.org/FAIRVASC#>
PREFIX bvas: <http://w3id.org/BVAS#>

SELECT (COUNT(DISTINCT ?patient) AS ?gpa_n)

WHERE {
?patient a fvc:Patient.
?patient fvc:hasPatientOverview ?PatientOverview.
?PatientOverview fvc:hasDiagnosis ?Diagnosis.
?Diagnosis fvc:mainDiagnosis ?maindiagnosis.
FILTER (?maindiagnosis =
"http://identifiers.org/orphanet:900"^^<http://www.w3.org/2001/XMLSchema#anyURI>)
}

# Query 4b: Number of MPA patients in the registry

PREFIX fvc: <http://w3id.org/FAIRVASC#>
PREFIX bvas: <http://w3id.org/BVAS#>

SELECT (COUNT(DISTINCT ?patient) AS ?mpa_n)

WHERE {
?patient a fvc:Patient.
?patient fvc:hasPatientOverview ?PatientOverview.
?PatientOverview fvc:hasDiagnosis ?Diagnosis.
?Diagnosis fvc:mainDiagnosis ?maindiagnosis.
FILTER (?maindiagnosis =
"http://identifiers.org/orphanet:727"^^<http://www.w3.org/2001/XMLSchema#anyURI>)
}

# Query 4c: Number of EGPA patients in the registry

PREFIX fvc: <http://w3id.org/FAIRVASC#>
PREFIX bvas: <http://w3id.org/BVAS#>

SELECT (COUNT(DISTINCT ?patient) AS ?egpa_n)

WHERE {
?patient a fvc:Patient.
?patient fvc:hasPatientOverview ?PatientOverview.
?PatientOverview fvc:hasDiagnosis ?Diagnosis.
```

```
?Diagnosis fvc:mainDiagnosis ?maindiagnosis.
FILTER (?maindiagnosis =
"http://identifiers.org/orphanet:183"^^<http://www.w3.org/2001/XMLSchema#anyURI>)
}

# Query 4d: Number of unspecified AAV patients in the registry

PREFIX fvc: <http://w3id.org/FAIRVASC#>
PREFIX bvas: <http://w3id.org/BVAS#>

SELECT (COUNT(DISTINCT ?patient) AS ?uns_AAV_n)

WHERE {
?patient a fvc:Patient.
?patient fvc:hasPatientOverview ?PatientOverview.
?PatientOverview fvc:hasDiagnosis ?Diagnosis.
?Diagnosis fvc:mainDiagnosis ?maindiagnosis.
FILTER (?maindiagnosis =
"http://identifiers.org/orphanet:156152"^^<http://www.w3.org/2001/XMLSchema#anyURI
>)
}


# Query 5a: Number of PR3 positive patients in the registry

PREFIX fvc: <http://w3id.org/FAIRVASC#>
PREFIX bvas: <http://w3id.org/BVAS#>

SELECT (COUNT(DISTINCT ?patient) AS ?pr3_n)

WHERE {
?patient a fvc:Patient.
?patient fvc:hasPatientOverview ?PatientOverview.
?PatientOverview fvc:hasANCA ?ANCA.
?ANCA fvc:ancaSpec ?elisa_anca.

  FILTER (?elisa_anca = "PR3 positive")
}


# Query 5b: Number of MPO positive patients in the registry

PREFIX fvc: <http://w3id.org/FAIRVASC#>
PREFIX bvas: <http://w3id.org/BVAS#>

SELECT (COUNT(DISTINCT ?patient) AS ?mpo_n)
```

```
WHERE {
?patient a fvc:Patient.
?patient fvc:hasPatientOverview ?PatientOverview.
?PatientOverview fvc:hasANCA ?ANCA.
?ANCA fvc:ancaSpec ?elisa_anca.

  FILTER (?elisa_anca = "MPO positive")
}

# Query 5c: Number of ELISA negative patients in the registry

PREFIX fvc: <http://w3id.org/FAIRVASC#>
PREFIX bvas: <http://w3id.org/BVAS#>

SELECT (COUNT(DISTINCT ?patient) AS ?negative_n)

WHERE {
?patient a fvc:Patient.
?patient fvc:hasPatientOverview ?PatientOverview.
?PatientOverview fvc:hasANCA ?ANCA.
?ANCA fvc:ancaSpec ?elisa_anca.

  FILTER (?elisa_anca = "ELISA negative")
}

# Query 5d: Number of PR3 and MPO positive patients in the registry

PREFIX fvc: <http://w3id.org/FAIRVASC#>
PREFIX bvas: <http://w3id.org/BVAS#>

SELECT (COUNT(DISTINCT ?patient) AS ?double_n)

WHERE {
?patient a fvc:Patient.
?patient fvc:hasPatientOverview ?PatientOverview.
?PatientOverview fvc:hasANCA ?ANCA.
?ANCA fvc:ancaSpec ?elisa_anca.

  FILTER (?elisa_anca = "MPO and PR3 positive")
}


# Query 6:

# Query 6a: Number of patients with constitutional or muskeloskelatal inolvement in the
registry
```

```
PREFIX fvc: <http://w3id.org/FAIRVASC#>
PREFIX bvas: <http://w3id.org/BVAS#>

SELECT (COUNT(DISTINCT ?patient) AS ?general_n)

WHERE{

?patient a fvc:Patient .
?patient fvc:hasPatientOverview ?PatientOverview.
?PatientOverview fvc:hasOrganPattern ?OrganPattern.
?OrganPattern fvc:affectedOrgan ?organ.
?OrganPattern fvc:genericTerm ?generic.

  FILTER (?organ = "Constitutional" || ?organ = "Muscoskeletal")
  FILTER (?generic = "true"^^<http://www.w3.org/2001/XMLSchema#boolean>)
}
```

```
# Query 6b: Number of patients with mucous membrane, cutaneous or eye involvement in the registry

PREFIX fvc: <http://w3id.org/FAIRVASC#>
PREFIX bvas: <http://w3id.org/BVAS#>

SELECT (COUNT(DISTINCT ?patient) AS ?muc_cut_eye_n)

WHERE{

?patient a fvc:Patient .
?patient fvc:hasPatientOverview ?PatientOverview.
?PatientOverview fvc:hasOrganPattern ?OrganPattern.
?OrganPattern fvc:affectedOrgan ?organ.
?OrganPattern fvc:genericTerm ?generic.

  FILTER (?organ = "MucocutaneousEye")
  FILTER (?generic = "true"^^<http://www.w3.org/2001/XMLSchema#boolean>)
}
```

```
# Query 6c: Number of patients with ENT involvement in the registry

PREFIX fvc: <http://w3id.org/FAIRVASC#>
PREFIX bvas: <http://w3id.org/BVAS#>

SELECT (COUNT(DISTINCT ?patient) AS ?ent_n)
```

```
WHERE{

?patient a fvc:Patient .
?patient fvc:hasPatientOverview ?PatientOverview.
?PatientOverview fvc:hasOrganPattern ?OrganPattern.
?OrganPattern fvc:affectedOrgan ?organ.
?OrganPattern fvc:genericTerm ?generic.

  FILTER (?organ = "Ear/Nose/Throat")
  FILTER (?generic = "true"^^<http://www.w3.org/2001/XMLSchema#boolean>)
}

# Query 6d: Number of patients with lung involvement in the registry

PREFIX fvc: <http://w3id.org/FAIRVASC#>
PREFIX bvas: <http://w3id.org/BVAS#>

SELECT (COUNT(DISTINCT ?patient) AS ?lung_n)

WHERE{

?patient a fvc:Patient .
?patient fvc:hasPatientOverview ?PatientOverview.
?PatientOverview fvc:hasOrganPattern ?OrganPattern.
?OrganPattern fvc:affectedOrgan ?organ.
?OrganPattern fvc:genericTerm ?generic.

  FILTER (?organ = "Lung")
  FILTER (?generic = "true"^^<http://www.w3.org/2001/XMLSchema#boolean>)
}

# Query 6e: Number of patients with cardiovascular involvement in the registry

PREFIX fvc: <http://w3id.org/FAIRVASC#>
PREFIX bvas: <http://w3id.org/BVAS#>

SELECT (COUNT(DISTINCT ?patient) AS ?cvs_n)

WHERE{

?patient a fvc:Patient .
?patient fvc:hasPatientOverview ?PatientOverview.
?PatientOverview fvc:hasOrganPattern ?OrganPattern.
?OrganPattern fvc:affectedOrgan ?organ.
?OrganPattern fvc:genericTerm ?generic.

  FILTER (?organ = "Cardiovascular")
```

```
  FILTER (?generic = "true"^^<http://www.w3.org/2001/XMLSchema#boolean>)
}

# Query 6f: Number of patients with abdominal involvement in the registry

PREFIX fvc: <http://w3id.org/FAIRVASC#>
PREFIX bvas: <http://w3id.org/BVAS#>

SELECT (COUNT(DISTINCT ?patient) AS ?abdominal_n)

WHERE{

?patient a fvc:Patient .
?patient fvc:hasPatientOverview ?PatientOverview.
?PatientOverview fvc:hasOrganPattern ?OrganPattern.
?OrganPattern fvc:affectedOrgan ?organ.
?OrganPattern fvc:genericTerm ?generic.

  FILTER (?organ = "Abdominal")
  FILTER (?generic = "true"^^<http://www.w3.org/2001/XMLSchema#boolean>)
}


# Query 6g: Number of patients with kidney involvement in the registry

PREFIX fvc: <http://w3id.org/FAIRVASC#>
PREFIX bvas: <http://w3id.org/BVAS#>

SELECT (COUNT(DISTINCT ?patient) AS ?kidney_n)

WHERE{

?patient a fvc:Patient .
?patient fvc:hasPatientOverview ?PatientOverview.
?PatientOverview fvc:hasOrganPattern ?OrganPattern.
?OrganPattern fvc:affectedOrgan ?organ.
?OrganPattern fvc:genericTerm ?generic.

  FILTER (?organ = "Kidney")
  FILTER (?generic = "true"^^<http://www.w3.org/2001/XMLSchema#boolean>)
}

# Query 6h: Number of patients with nervous system involvement in the registry

PREFIX fvc: <http://w3id.org/FAIRVASC#>
PREFIX bvas: <http://w3id.org/BVAS#>
```

```
SELECT (COUNT(DISTINCT ?patient) AS ?nervous_n)

WHERE{

?patient a fvc:Patient .
?patient fvc:hasPatientOverview ?PatientOverview.
?PatientOverview fvc:hasOrganPattern ?OrganPattern.
?OrganPattern fvc:affectedOrgan ?organ.
?OrganPattern fvc:genericTerm ?generic.

  FILTER (?organ = "Nervous system")
  FILTER (?generic = "true"^^<http://www.w3.org/2001/XMLSchema#boolean>)
}

# Query 6h: Number of patients with organ pattern recorded in registry

PREFIX fvc: <http://w3id.org/FAIRVASC#>
PREFIX bvas: <http://w3id.org/BVAS#>

SELECT (COUNT(DISTINCT ?patient) AS ?n_organ_pattern)

WHERE{

?patient a fvc:Patient .
?patient fvc:hasPatientOverview ?PatientOverview.
?PatientOverview fvc:hasOrganPattern ?OrganPattern.
?OrganPattern fvc:affectedOrgan ?organ.
?OrganPattern fvc:genericTerm ?generic.
}


# Query 7a: Number of patients receiving PO CYC as induction treatment in the registry

PREFIX fvc: <http://w3id.org/FAIRVASC#>
PREFIX bvas: <http://w3id.org/BVAS#>

SELECT (COUNT(DISTINCT ?patient) AS ?PO_CYC_n)

WHERE {
?patient a fvc:Patient.
?patient fvc:hasPatientOverview ?PatientOverview.
?PatientOverview fvc:hasTreatment ?Treatment.
?Treatment a fvc:TreatmentInduction.
?Treatment fvc:treatmentType ?type.
?Treatment fvc:routeToAdministration ?route.
```

```
  FILTER (?type =
"http://identifiers.org/ncit:C405"^^<http://www.w3.org/2001/XMLSchema#anyURI>)
  FILTER (?route =
"http://identifiers.org/ncit:C38288"^^<http://www.w3.org/2001/XMLSchema#anyURI>)
}

# Query 7b: Number of patients receiving IV CYC as induction treatment in the registry

PREFIX fvc: <http://w3id.org/FAIRVASC#>
PREFIX bvas: <http://w3id.org/BVAS#>

SELECT (COUNT(DISTINCT ?patient) AS ?IV_CYC_n)

WHERE {
?patient a fvc:Patient.
?patient fvc:hasPatientOverview ?PatientOverview.
?PatientOverview fvc:hasTreatment ?Treatment.
?Treatment a fvc:TreatmentInduction.
?Treatment fvc:treatmentType ?type.
?Treatment fvc:routeToAdministration ?route.

  FILTER (?type =
"http://identifiers.org/ncit:C405"^^<http://www.w3.org/2001/XMLSchema#anyURI>)
  FILTER (?route =
"http://identifiers.org/ncit:C38276"^^<http://www.w3.org/2001/XMLSchema#anyURI>)
}


# Query 7c: Number of patients receiving RTX as induction treatment in the registry

PREFIX fvc: <http://w3id.org/FAIRVASC#>
PREFIX bvas: <http://w3id.org/BVAS#>

SELECT (COUNT(DISTINCT ?patient) AS ?RTX_n)

WHERE {
?patient a fvc:Patient.
?patient fvc:hasPatientOverview ?PatientOverview.
?PatientOverview fvc:hasTreatment ?Treatment.
?Treatment a fvc:TreatmentInduction.
?Treatment fvc:treatmentType ?type.

  FILTER (?type =
"http://identifiers.org/ncit:C1702"^^<http://www.w3.org/2001/XMLSchema#anyURI>)
}

# Query 7d: Number of patients with induction treatment in the registry
```

```
PREFIX fvc: <http://w3id.org/FAIRVASC#>
PREFIX bvas: <http://w3id.org/BVAS#>

SELECT (COUNT(DISTINCT ?patient) AS ?n_induction)

WHERE {
?patient a fvc:Patient.
?patient fvc:hasPatientOverview ?PatientOverview.
?PatientOverview fvc:hasTreatment ?Treatment.
?Treatment a fvc:TreatmentInduction.
?Treatment fvc:treatmentType ?type.

}


# Query 8a: Number of patients receiving AZA as maintenance treatment in the registry

PREFIX fvc: <http://w3id.org/FAIRVASC#>
PREFIX bvas: <http://w3id.org/BVAS#>

SELECT (COUNT(DISTINCT ?patient) AS ?AZA_n)

WHERE {
?patient a fvc:Patient.
?patient fvc:hasPatientOverview ?PatientOverview.
?PatientOverview fvc:hasTreatment ?Treatment.
?Treatment a fvc:TreatmentMaintenance.
?Treatment fvc:treatmentType ?type.

  FILTER (?type =
"http://identifiers.org/ncit:C290"^^<http://www.w3.org/2001/XMLSchema#anyURI>)
}


# Query 8b: Number of patients receiving RTX as maintenance treatment in the registry

PREFIX fvc: <http://w3id.org/FAIRVASC#>
PREFIX bvas: <http://w3id.org/BVAS#>

SELECT (COUNT(DISTINCT ?patient) AS ?RTX_n)

WHERE {
?patient a fvc:Patient.
?patient fvc:hasPatientOverview ?PatientOverview.
?PatientOverview fvc:hasTreatment ?Treatment.
?Treatment a fvc:TreatmentMaintenance.
```

```
?Treatment fvc:treatmentType ?type.

  FILTER (?type =
"http://identifiers.org/ncit:C1702"^^<http://www.w3.org/2001/XMLSchema#anyURI>)
}


# Query 8c: Number of patients with maintenance treatment in the registry

PREFIX fvc: <http://w3id.org/FAIRVASC#>
PREFIX bvas: <http://w3id.org/BVAS#>

SELECT (COUNT(DISTINCT ?patient) AS ?n_maintenance)

WHERE {
?patient a fvc:Patient.
?patient fvc:hasPatientOverview ?PatientOverview.
?PatientOverview fvc:hasTreatment ?Treatment.
?Treatment a fvc:TreatmentMaintenance.
?Treatment fvc:treatmentType ?type.

}

# Query 9a: Number of patients dying in the registry

PREFIX fvc: <http://w3id.org/FAIRVASC#>
PREFIX bvas: <http://w3id.org/BVAS#>

SELECT (COUNT(DISTINCT ?patient) AS ?dead_n)

WHERE {
?patient a fvc:Patient.
?patient fvc:hasClinicalOutcome ?ClinicalOutcomes.
?ClinicalOutcomes fvc:death ?death.

FILTER (?death = "true"^^<http://www.w3.org/2001/XMLSchema#boolean>)
}

# Query 9b: Number of patients reaching ESKD in the registry

PREFIX fvc: <http://w3id.org/FAIRVASC#>
PREFIX bvas: <http://w3id.org/BVAS#>

SELECT (COUNT(DISTINCT ?patient) AS ?eskd_n)

WHERE {
?patient a fvc:Patient.
```

```
?patient fvc:hasClinicalOutcome ?ClinicalOutcomes.
?ClinicalOutcomes fvc:ESKDApproximation ?eskdapprox.

FILTER (?eskdapprox = "true"^^<http://www.w3.org/2001/XMLSchema#boolean>)
}
```

```
# Query 9c: Mean time of follow up in the registry

PREFIX fvc: <http://w3id.org/FAIRVASC#>
PREFIX bvas: <http://w3id.org/BVAS#>
PREFIX xsd: <http://www.w3.org/2001/XMLSchema#>

SELECT (AVG(xsd:integer(?followuptime)/365) AS ?avg_followuptime_years)

WHERE {
?patient a fvc:Patient.
?patient fvc:hasPatientOverview ?PatientOverview.
?PatientOverview fvc:hasDiagnosis ?Diagnosis.
?Diagnosis fvc:dateOfDiagnosis ?dateofdiagnosis.
?patient fvc:hasClinicalOutcome ?ClinicalOutcomes.
?ClinicalOutcomes fvc:lastRecordedContact ?dateoffollowup.
  BIND(
  (((YEAR(?dateoffollowup))-YEAR(?dateofdiagnosis))*365) +
    ((MONTH(?dateoffollowup) - MONTH(?dateofdiagnosis))*12) +
    (DAY(?dateoffollowup) - DAY(?dateofdiagnosis))
  AS ?followuptime)

}
```

```
# Query 9d: Follow-up time variance in registry in years (this has to be sqrt to retrieve
standard deviation of follow-up time which can't be done in pure SPARQL)

PREFIX fvc: <http://w3id.org/FAIRVASC#>
PREFIX bvas: <http://w3id.org/BVAS#>
PREFIX xsd: <http://www.w3.org/2001/XMLSchema#>

SELECT(SUM(((xsd:integer(?followuptime)/365)-
?avg_followuptime_years)*((xsd:integer(?followuptime)/365)-
?avg_followuptime_years))/(COUNT(?patient) - 1) as ?variance)

WHERE {
?patient a fvc:Patient.
?patient fvc:hasPatientOverview ?PatientOverview.
?PatientOverview fvc:hasDiagnosis ?Diagnosis.
?Diagnosis fvc:dateOfDiagnosis ?dateofdiagnosis.
?patient fvc:hasClinicalOutcome ?ClinicalOutcomes.
```

```
?ClinicalOutcomes fvc:lastRecordedContact ?dateoffollowup.
  BIND(
  (((YEAR(?dateoffollowup))-YEAR(?dateofdiagnosis))*365) +
    ((MONTH(?dateoffollowup) - MONTH(?dateofdiagnosis))*12) +
    (DAY(?dateoffollowup) - DAY(?dateofdiagnosis))
  AS ?followuptime)
 {
SELECT (AVG(xsd:integer(?followuptime)/365) AS ?avg_followuptime_years)

WHERE {
?patient a fvc:Patient.
?patient fvc:hasPatientOverview ?PatientOverview.
?PatientOverview fvc:hasDiagnosis ?Diagnosis.
?Diagnosis fvc:dateOfDiagnosis ?dateofdiagnosis.
?patient fvc:hasClinicalOutcome ?ClinicalOutcomes.
?ClinicalOutcomes fvc:lastRecordedContact ?dateoffollowup.
  BIND(
  (((YEAR(?dateoffollowup))-YEAR(?dateofdiagnosis))*365) +
    ((MONTH(?dateoffollowup) - MONTH(?dateofdiagnosis))*12) +
    (DAY(?dateoffollowup) - DAY(?dateofdiagnosis))
  AS ?followuptime)

}
  }
}

# Query 9e: Sum of squared registry deviations from global mean follow up time

PREFIX fvc: <http://w3id.org/FAIRVASC#>
PREFIX bvas: <http://w3id.org/BVAS#>
PREFIX xsd: <http://www.w3.org/2001/XMLSchema#>

SELECT(SUM(((xsd:integer(?followuptime)/365)-6.2)*((xsd:integer(?followuptime)/365)-
6.2)) as ?reg_sumsquare)

WHERE {
?patient a fvc:Patient.
?patient fvc:hasPatientOverview ?PatientOverview.
?PatientOverview fvc:hasDiagnosis ?Diagnosis.
?Diagnosis fvc:dateOfDiagnosis ?dateofdiagnosis.
?patient fvc:hasClinicalOutcome ?ClinicalOutcomes.
?ClinicalOutcomes fvc:lastRecordedContact ?dateoffollowup.
  BIND(
  (((YEAR(?dateoffollowup))-YEAR(?dateofdiagnosis))*365) +
    ((MONTH(?dateoffollowup) - MONTH(?dateofdiagnosis))*12) +
    (DAY(?dateoffollowup) - DAY(?dateofdiagnosis))
  AS ?followuptime)
```

```
}


# Query 10a: Mean s-creatinine at diagnosis
PREFIX fvc: <http://w3id.org/FAIRVASC#>
PREFIX bvas: <http://w3id.org/BVAS#>

SELECT
(AVG(?crea) AS ?avg_crea)

WHERE {
?patient a fvc:Patient.
?patient fvc:hasPatientOverview ?PatientOverview.
?PatientOverview fvc:hasCreatinineAtDiagnosis ?creadiagnosis.
?creadiagnosis fvc:testValue ?crea.

}



# Query 10b: Mean s-creatinine at diagnosis availability

PREFIX fvc: <http://w3id.org/FAIRVASC#>
PREFIX bvas: <http://w3id.org/BVAS#>

SELECT (COUNT(DISTINCT ?patient) AS ?creadiagnosis_n)

WHERE {
?patient a fvc:Patient.
?patient fvc:hasPatientOverview ?PatientOverview.
?PatientOverview fvc:hasCreatinineAtDiagnosis ?creadiagnosis.

}


# Query 10c: Creatinine at diagnosis variance in registry (this has to be sqrt to retrieve
standard deviation (SD) of age which can't be done in pure SPARQL)

PREFIX fvc: <http://w3id.org/FAIRVASC#>
PREFIX bvas: <http://w3id.org/BVAS#>


SELECT(SUM((?crea-?avg_crea)*(?crea-?avg_crea))/(COUNT(?creadiagnosis) - 1) as
?variance)

WHERE {
?patient a fvc:Patient.
```

```
?patient fvc:hasPatientOverview ?PatientOverview.
?PatientOverview fvc:hasCreatinineAtDiagnosis ?creadiagnosis.
?creadiagnosis fvc:testValue ?crea.
 {

SELECT
(AVG(?crea) AS ?avg_crea)

WHERE {
?patient a fvc:Patient.
?patient fvc:hasPatientOverview ?PatientOverview.
?PatientOverview fvc:hasCreatinineAtDiagnosis ?creadiagnosis.
?creadiagnosis fvc:testValue ?crea.
}
  }
}

# Query 10d: Sum of squared registry deviations from global mean creatinine at diagnosis

PREFIX fvc: <http://w3id.org/FAIRVASC#>
PREFIX bvas: <http://w3id.org/BVAS#>


SELECT(SUM((?crea-198)*(?crea-198)) as ?reg_sumsquare)

WHERE {
?patient a fvc:Patient.
?patient fvc:hasPatientOverview ?PatientOverview.
?PatientOverview fvc:hasCreatinineAtDiagnosis ?creadiagnosis.
?creadiagnosis fvc:testValue ?crea.
}


# Query 11a: Event-rate of ESKD. This query calculates the number of events of ESKD in four
time intervals and the sum of total patient follow-up time in each time frame. End of follow
up is date of ESKD or date of last visit, whichever occurs first. It assumes good data quality,
no "negative" follow up time etc. All this is stratified per diagnosis. If no event is occurring in
any of the timeframes, the diagnosis will not show. It assumes that end-of-follow-up is at
date of death if occurring before.

PREFIX fvc: <http://w3id.org/FAIRVASC#>
PREFIX bvas: <http://w3id.org/BVAS#>
PREFIX xsd: <http://www.w3.org/2001/XMLSchema#>
PREFIX fn:   <http://www.w3.org/2005/xpath-functions#>

SELECT
(SUM(?personTime_1YR) as ?totalpersonTime_1YR)
```

```
(SUM(?personTime_1until2YR) as ?totalpersonTime_1until2YR)
(SUM(?personTime_2until5YR) as ?totalpersonTime_2until5YR)
(SUM(?personTime_after5YR) as ?totalpersonTime_after5YR)

(SUM(?sumDeaths_1YR) as ?totalsumESKD_1YR)
(SUM(?sumDeaths_1until2YR) as ?totalsumESKD_1until2YR)
(SUM(?sumDeaths_2until5YR) as ?totalsumESKD_2until5YR)
(SUM(?sumDeaths_5YR) as ?totalsumESKD_5YR)

?maindiagnosis

WHERE {
{
?patient a fvc:Patient_hold .
}
UNION
{
{
SELECT
((SUM(?TOTALTIME1YR)/COUNT(?patient)) AS ?personTime_1YR)
((SUM(?TOTALTIME1_2YR)/COUNT(?patient)) AS ?personTime_1until2YR)
((SUM(?TOTALTIME2_5YR)/COUNT(?patient)) AS ?personTime_2until5YR)
((SUM(?TOTALTIMEAFTER5YR)/COUNT(?patient))AS ?personTime_after5YR)

(SUM(?inf1yr) AS ?sumDeaths_1YR)
(SUM(?inf1until2yr) AS ?sumDeaths_1until2YR)
(SUM(?inf2until5yr)AS?sumDeaths_2until5YR)
(SUM(?infafter5yr)AS?sumDeaths_5YR)

?maindiagnosis

WHERE {
?patient a fvc:Patient;
fvc:hasClinicalOutcome ?clinicalOutcome;
fvc:hasPatientOverview ?patientOverview.
?patientOverview fvc:hasDiagnosis ?dia.
?clinicalOutcome fvc:lastRecordedContact ?lastRecordedContact;
fvc:dateOfESKD ?deathDate.
?dia fvc:dateOfDiagnosis ?dateOfDiagnosis.
?dia fvc:mainDiagnosis ?maindiagnosis.

BIND(xsd:integer(xsd:float(str(floor((fn:days-from-duration(?deathDate-
?dateOfDiagnosis)))))) AS ?timeDiagnosisDeath)
BIND(IF(?timeDiagnosisDeath <365.25, 1, 0) AS ?inf1yr)
BIND(IF(?timeDiagnosisDeath <730.5 && ?timeDiagnosisDeath >=365.25, 1, 0) AS
?inf1until2yr)
```

```
BIND(IF(?timeDiagnosisDeath <1826.25 && ?timeDiagnosisDeath >=730.5, 1, 0) AS
?inf2until5yr)
BIND(IF(?timeDiagnosisDeath >=1826.25, 1, 0) AS ?infafter5yr)
{
SELECT
(SUM(?personTimeDays_1YR)/365.25 AS ?TOTALTIME1YR)
(SUM(?personTimeDays_1until2YR)/365.25 AS ?TOTALTIME1_2YR)
(SUM(?personTimeDays_2until5YR)/365.25 AS ?TOTALTIME2_5YR)
(SUM(?personTimeDays_after5YR)/365.25 AS ?TOTALTIMEAFTER5YR)
?maindiagnosis


WHERE {
?patient a fvc:Patient;
fvc:hasClinicalOutcome ?clinicalOutcome;
fvc:hasPatientOverview ?patientOverview.
?clinicalOutcome fvc:lastRecordedContact ?lastRecordedContact;
fvc:ESKDApproximation ?hasDied.
?patientOverview fvc:hasDiagnosis ?dia.
?dia fvc:mainDiagnosis ?maindiagnosis.

?dia fvc:dateOfDiagnosis ?dateOfDiagnosis .
OPTIONAL{ ?clinicalOutcome fvc:dateOfESKD ?dateDeath . }
BIND("1000-01-01T00:00:00"^^xsd:dateTime as ?defaultDateOfDeath)
BIND(coalesce(?dateDeath, ?defaultDateOfDeath) as ?dateOfDeath)
BIND(IF(?dateOfDeath > ?lastRecordedContact, ?dateOfDeath, ?lastRecordedContact) AS
?lastDate)
BIND(xsd:integer(xsd:float(str(floor((fn:days-from-duration(?lastDate- ?dateOfDiagnosis))))))
AS ?patientTotalTime)
BIND(IF(?patientTotalTime >= 365.25, 365.25, ?patientTotalTime) AS ?personTimeDays_1YR)
BIND(IF(?patientTotalTime >= 730.5, 730.5, ?patientTotalTime) AS ?personTimeDays_2YR)
BIND(IF(?patientTotalTime >= 1826.25, 1826.25, ?patientTotalTime) AS
?personTimeDays_5YR)
BIND(?personTimeDays_2YR - ?personTimeDays_1YR AS ?personTimeDays_1until2YR)
BIND(?personTimeDays_5YR - ?personTimeDays_2YR AS ?personTimeDays_2until5YR)
BIND(?patientTotalTime - ?personTimeDays_5YR AS ?personTimeDays_after5YR)
}
GROUP BY ?maindiagnosis
}
}
GROUP BY ?maindiagnosis
}
}
}
GROUP BY ?maindiagnosis
```

```
# Query 11b: Event-rate of ESKD. This query returns the follow up time for ESKD rates even if
the number of events in one diagnosis group is zero

PREFIX fvc: <http://w3id.org/FAIRVASC#>
PREFIX bvas: <http://w3id.org/BVAS#>
PREFIX xsd: <http://www.w3.org/2001/XMLSchema#>
PREFIX fn:   <http://www.w3.org/2005/xpath-functions#>

SELECT
(SUM(?personTime_1YR) as ?totalpersonTime_1YR)
(SUM(?personTime_1until2YR) as ?totalpersonTime_1until2YR)
(SUM(?personTime_2until5YR) as ?totalpersonTime_2until5YR)
(SUM(?personTime_after5YR) as ?totalpersonTime_after5YR)

?maindiagnosis

WHERE {
{
?patient a fvc:Patient_hold .
}
UNION
{
{
SELECT
((SUM(?TOTALTIME1YR)/COUNT(?patient)) AS ?personTime_1YR)
((SUM(?TOTALTIME1_2YR)/COUNT(?patient)) AS ?personTime_1until2YR)
((SUM(?TOTALTIME2_5YR)/COUNT(?patient)) AS ?personTime_2until5YR)
((SUM(?TOTALTIMEAFTER5YR)/COUNT(?patient))AS ?personTime_after5YR)

(SUM(?inf1yr) AS ?sumDeaths_1YR)
(SUM(?inf1until2yr) AS ?sumDeaths_1until2YR)
(SUM(?inf2until5yr)AS?sumDeaths_2until5YR)
(SUM(?infafter5yr)AS?sumDeaths_5YR)

?maindiagnosis

WHERE {
?patient a fvc:Patient;
fvc:hasClinicalOutcome ?clinicalOutcome;
fvc:hasPatientOverview ?patientOverview.
?patientOverview fvc:hasDiagnosis ?dia.
?clinicalOutcome fvc:lastRecordedContact ?lastRecordedContact.
	OPTIONAL {?clinicalOutcome fvc:dateOfESKD ?deathDate.}
?dia fvc:dateOfDiagnosis ?dateOfDiagnosis.
?dia fvc:mainDiagnosis ?maindiagnosis.
```

```
BIND(xsd:integer(xsd:float(str(floor((fn:days-from-duration(?deathDate-
?dateOfDiagnosis)))))) AS ?timeDiagnosisDeath)
BIND(IF(?timeDiagnosisDeath <365.25, 1, 0) AS ?inf1yr)
BIND(IF(?timeDiagnosisDeath <730.5 && ?timeDiagnosisDeath >=365.25, 1, 0) AS
?inf1until2yr)
BIND(IF(?timeDiagnosisDeath <1826.25 && ?timeDiagnosisDeath >=730.5, 1, 0) AS
?inf2until5yr)
BIND(IF(?timeDiagnosisDeath >=1826.25, 1, 0) AS ?infafter5yr)
{
SELECT
(SUM(?personTimeDays_1YR)/365.25 AS ?TOTALTIME1YR)
(SUM(?personTimeDays_1until2YR)/365.25 AS ?TOTALTIME1_2YR)
(SUM(?personTimeDays_2until5YR)/365.25 AS ?TOTALTIME2_5YR)
(SUM(?personTimeDays_after5YR)/365.25 AS ?TOTALTIMEAFTER5YR)
?maindiagnosis


WHERE {
?patient a fvc:Patient;
fvc:hasClinicalOutcome ?clinicalOutcome;
fvc:hasPatientOverview ?patientOverview.
?clinicalOutcome fvc:lastRecordedContact ?lastRecordedContact.
 ?clinicalOutcome fvc:ESKDApproximation ?hasDied.
?patientOverview fvc:hasDiagnosis ?dia.
?dia fvc:mainDiagnosis ?maindiagnosis.

?dia fvc:dateOfDiagnosis ?dateOfDiagnosis .
OPTIONAL{ ?clinicalOutcome fvc:dateOfESKD ?dateDeath . }
BIND("1000-01-01T00:00:00"^^xsd:dateTime as ?defaultDateOfDeath)
BIND(coalesce(?dateDeath, ?defaultDateOfDeath) as ?dateOfDeath)
BIND(IF(?dateOfDeath > ?lastRecordedContact, ?dateOfDeath, ?lastRecordedContact) AS
?lastDate)
BIND(xsd:integer(xsd:float(str(floor((fn:days-from-duration(?lastDate- ?dateOfDiagnosis))))))
AS ?patientTotalTime)
BIND(IF(?patientTotalTime >= 365.25, 365.25, ?patientTotalTime) AS ?personTimeDays_1YR)
BIND(IF(?patientTotalTime >= 730.5, 730.5, ?patientTotalTime) AS ?personTimeDays_2YR)
BIND(IF(?patientTotalTime >= 1826.25, 1826.25, ?patientTotalTime) AS
?personTimeDays_5YR)
BIND(?personTimeDays_2YR - ?personTimeDays_1YR AS ?personTimeDays_1until2YR)
BIND(?personTimeDays_5YR - ?personTimeDays_2YR AS ?personTimeDays_2until5YR)
BIND(?patientTotalTime - ?personTimeDays_5YR AS ?personTimeDays_after5YR)
}
GROUP BY ?maindiagnosis
}
}
GROUP BY ?maindiagnosis
}
```

```
}
}
GROUP BY ?maindiagnosis
```

Query 12: Event-rate of mortality. This query calculates the number of events of death in four time intervals and the sum of total patient follow-up time in each time frame. End of follow up is date of death or date of last visit, whichever occurs first. It assumes good data quality, no "negative" follow up time etc. All this is stratified per diagnosis. If no event is occurring in any of the timeframes, the diagnosis will not show.

```
PREFIX fvc: <http://w3id.org/FAIRVASC#>
PREFIX bvas: <http://w3id.org/BVAS#>
PREFIX xsd: <http://www.w3.org/2001/XMLSchema#>
PREFIX fn:   <http://www.w3.org/2005/xpath-functions#>

SELECT
(SUM(?personTime_1YR) as ?totalpersonTime_1YR)
(SUM(?personTime_1until2YR) as ?totalpersonTime_1until2YR)
(SUM(?personTime_2until5YR) as ?totalpersonTime_2until5YR)
(SUM(?personTime_after5YR) as ?totalpersonTime_after5YR)

(SUM(?sumDeaths_1YR) as ?totalsumDeaths_1YR)
(SUM(?sumDeaths_1until2YR) as ?totalsumDeaths_1until2YR)
(SUM(?sumDeaths_2until5YR) as ?totalsumDeaths_2until5YR)
(SUM(?sumDeaths_5YR) as ?totalsumDeaths_5YR)

?maindiagnosis

WHERE {
{
?patient a fvc:Patient_hold .
}
UNION
{
{
SELECT
((SUM(?TOTALTIME1YR)/COUNT(?patient)) AS ?personTime_1YR)
((SUM(?TOTALTIME1_2YR)/COUNT(?patient)) AS ?personTime_1until2YR)
((SUM(?TOTALTIME2_5YR)/COUNT(?patient)) AS ?personTime_2until5YR)
((SUM(?TOTALTIMEAFTER5YR)/COUNT(?patient))AS ?personTime_after5YR)

(SUM(?inf1yr) AS ?sumDeaths_1YR)
(SUM(?inf1until2yr) AS ?sumDeaths_1until2YR)
(SUM(?inf2until5yr)AS?sumDeaths_2until5YR)
```

```
(SUM(?infafter5yr)AS?sumDeaths_5YR)

?maindiagnosis

WHERE {
?patient a fvc:Patient;
fvc:hasClinicalOutcome ?clinicalOutcome;
fvc:hasPatientOverview ?patientOverview.
?patientOverview fvc:hasDiagnosis ?dia.
?clinicalOutcome fvc:lastRecordedContact ?lastRecordedContact;
fvc:dateOfDeath ?deathDate.
?dia fvc:dateOfDiagnosis ?dateOfDiagnosis.
?dia fvc:mainDiagnosis ?maindiagnosis.

BIND(xsd:integer(xsd:float(str(floor((fn:days-from-duration(?deathDate-
?dateOfDiagnosis)))))) AS ?timeDiagnosisDeath)
BIND(IF(?timeDiagnosisDeath <365.25, 1, 0) AS ?inf1yr)
BIND(IF(?timeDiagnosisDeath <730.5 && ?timeDiagnosisDeath >=365.25, 1, 0) AS
?inf1until2yr)
BIND(IF(?timeDiagnosisDeath <1826.25 && ?timeDiagnosisDeath >=730.5, 1, 0) AS
?inf2until5yr)
BIND(IF(?timeDiagnosisDeath >=1826.25, 1, 0) AS ?infafter5yr)
{
SELECT
(SUM(?personTimeDays_1YR)/365.25 AS ?TOTALTIME1YR)
(SUM(?personTimeDays_1until2YR)/365.25 AS ?TOTALTIME1_2YR)
(SUM(?personTimeDays_2until5YR)/365.25 AS ?TOTALTIME2_5YR)
(SUM(?personTimeDays_after5YR)/365.25 AS ?TOTALTIMEAFTER5YR)
?maindiagnosis


WHERE {
?patient a fvc:Patient;
fvc:hasClinicalOutcome ?clinicalOutcome;
fvc:hasPatientOverview ?patientOverview.
?clinicalOutcome fvc:lastRecordedContact ?lastRecordedContact;
fvc:death ?hasDied.
?patientOverview fvc:hasDiagnosis ?dia.
?dia fvc:mainDiagnosis ?maindiagnosis.

?dia fvc:dateOfDiagnosis ?dateOfDiagnosis .
OPTIONAL{ ?clinicalOutcome fvc:dateOfDeath ?dateDeath . }
BIND("1000-01-01T00:00:00"^^xsd:dateTime as ?defaultDateOfDeath)
BIND(coalesce(?dateDeath, ?defaultDateOfDeath) as ?dateOfDeath)
BIND(IF(?dateOfDeath > ?lastRecordedContact, ?dateOfDeath, ?lastRecordedContact) AS
?lastDate)
```

```
BIND(xsd:integer(xsd:float(str(floor((fn:days-from-duration(?lastDate- ?dateOfDiagnosis))))))
AS ?patientTotalTime)
BIND(IF(?patientTotalTime >= 365.25, 365.25, ?patientTotalTime) AS ?personTimeDays_1YR)
BIND(IF(?patientTotalTime >= 730.5, 730.5, ?patientTotalTime) AS ?personTimeDays_2YR)
BIND(IF(?patientTotalTime >= 1826.25, 1826.25, ?patientTotalTime) AS
?personTimeDays_5YR)
BIND(?personTimeDays_2YR - ?personTimeDays_1YR AS ?personTimeDays_1until2YR)
BIND(?personTimeDays_5YR - ?personTimeDays_2YR AS ?personTimeDays_2until5YR)
BIND(?patientTotalTime - ?personTimeDays_5YR AS ?personTimeDays_after5YR)
}
GROUP BY ?maindiagnosis
}
}
GROUP BY ?maindiagnosis
}
}
}
GROUP BY ?maindiagnosis


# Query 12b: Mortality-rate. This query returns the follow up time for mortality rates even if
the number of events in one diagnosis group is zero
PREFIX fvc: <http://w3id.org/FAIRVASC#>
PREFIX bvas: <http://w3id.org/BVAS#>
PREFIX xsd: <http://www.w3.org/2001/XMLSchema#>
PREFIX fn:   <http://www.w3.org/2005/xpath-functions#>

SELECT
(SUM(?personTime_1YR) as ?totalpersonTime_1YR)
(SUM(?personTime_1until2YR) as ?totalpersonTime_1until2YR)
(SUM(?personTime_2until5YR) as ?totalpersonTime_2until5YR)
(SUM(?personTime_after5YR) as ?totalpersonTime_after5YR)

?maindiagnosis

WHERE {
{
?patient a fvc:Patient_hold .
}
UNION
{
{
SELECT
((SUM(?TOTALTIME1YR)/COUNT(?patient)) AS ?personTime_1YR)
((SUM(?TOTALTIME1_2YR)/COUNT(?patient)) AS ?personTime_1until2YR)
((SUM(?TOTALTIME2_5YR)/COUNT(?patient)) AS ?personTime_2until5YR)
((SUM(?TOTALTIMEAFTER5YR)/COUNT(?patient))AS ?personTime_after5YR)
```

```
(SUM(?inf1yr) AS ?sumDeaths_1YR)
(SUM(?inf1until2yr) AS ?sumDeaths_1until2YR)
(SUM(?inf2until5yr)AS?sumDeaths_2until5YR)
(SUM(?infafter5yr)AS?sumDeaths_5YR)

?maindiagnosis

WHERE {
?patient a fvc:Patient;
fvc:hasClinicalOutcome ?clinicalOutcome;
fvc:hasPatientOverview ?patientOverview.
?patientOverview fvc:hasDiagnosis ?dia.
?clinicalOutcome fvc:lastRecordedContact ?lastRecordedContact.
	OPTIONAL {?clinicalOutcome fvc:dateOfDeath ?deathDate.}
?dia fvc:dateOfDiagnosis ?dateOfDiagnosis.
?dia fvc:mainDiagnosis ?maindiagnosis.

BIND(xsd:integer(xsd:float(str(floor((fn:days-from-duration(?deathDate-
?dateOfDiagnosis)))))) AS ?timeDiagnosisDeath)
BIND(IF(?timeDiagnosisDeath <365.25, 1, 0) AS ?inf1yr)
BIND(IF(?timeDiagnosisDeath <730.5 && ?timeDiagnosisDeath >=365.25, 1, 0) AS
?inf1until2yr)
BIND(IF(?timeDiagnosisDeath <1826.25 && ?timeDiagnosisDeath >=730.5, 1, 0) AS
?inf2until5yr)
BIND(IF(?timeDiagnosisDeath >=1826.25, 1, 0) AS ?infafter5yr)
{
SELECT
(SUM(?personTimeDays_1YR)/365.25 AS ?TOTALTIME1YR)
(SUM(?personTimeDays_1until2YR)/365.25 AS ?TOTALTIME1_2YR)
(SUM(?personTimeDays_2until5YR)/365.25 AS ?TOTALTIME2_5YR)
(SUM(?personTimeDays_after5YR)/365.25 AS ?TOTALTIMEAFTER5YR)
?maindiagnosis


WHERE {
?patient a fvc:Patient;
fvc:hasClinicalOutcome ?clinicalOutcome;
fvc:hasPatientOverview ?patientOverview.
?clinicalOutcome fvc:lastRecordedContact ?lastRecordedContact;
fvc:death ?hasDied.
?patientOverview fvc:hasDiagnosis ?dia.
?dia fvc:mainDiagnosis ?maindiagnosis.

?dia fvc:dateOfDiagnosis ?dateOfDiagnosis .
OPTIONAL{ ?clinicalOutcome fvc:dateOfDeath ?dateDeath . }
BIND("1000-01-01T00:00:00"^^xsd:dateTime as ?defaultDateOfDeath)
```

```
BIND(coalesce(?dateDeath, ?defaultDateOfDeath) as ?dateOfDeath)
BIND(IF(?dateOfDeath > ?lastRecordedContact, ?dateOfDeath, ?lastRecordedContact) AS
?lastDate)
BIND(xsd:integer(xsd:float(str(floor((fn:days-from-duration(?lastDate- ?dateOfDiagnosis))))))
AS ?patientTotalTime)
BIND(IF(?patientTotalTime >= 365.25, 365.25, ?patientTotalTime) AS ?personTimeDays_1YR)
BIND(IF(?patientTotalTime >= 730.5, 730.5, ?patientTotalTime) AS ?personTimeDays_2YR)
BIND(IF(?patientTotalTime >= 1826.25, 1826.25, ?patientTotalTime) AS
?personTimeDays_5YR)
BIND(?personTimeDays_2YR - ?personTimeDays_1YR AS ?personTimeDays_1until2YR)
BIND(?personTimeDays_5YR - ?personTimeDays_2YR AS ?personTimeDays_2until5YR)
BIND(?patientTotalTime - ?personTimeDays_5YR AS ?personTimeDays_after5YR)
}
GROUP BY ?maindiagnosis
}
}
GROUP BY ?maindiagnosis
}
}
}
GROUP BY ?maindiagnosis
```

Query 12.2a: UPDATED. Event-rate of mortality. This query calculates the number of events of death in four time intervals and the sum of total patient follow-up time in each time frame. End of follow up is date of death or date of last visit, where date of death always takes precedence. It assumes good data quality, no "negative" follow up time etc. All this is stratified per diagnosis. If no event is occurring in any of the timeframes, the diagnosis will not show.

```
PREFIX fvc: <http://w3id.org/FAIRVASC#>
PREFIX bvas: <http://w3id.org/BVAS#>
PREFIX xsd: <http://www.w3.org/2001/XMLSchema#>
PREFIX fn:   <http://www.w3.org/2005/xpath-functions#>

SELECT
(SUM(?personTime_1YR) as ?totalpersonTime_1YR)
(SUM(?personTime_1until2YR) as ?totalpersonTime_1until2YR)
(SUM(?personTime_2until5YR) as ?totalpersonTime_2until5YR)
(SUM(?personTime_after5YR) as ?totalpersonTime_after5YR)

(SUM(?sumDeaths_1YR) as ?totalsumDeaths_1YR)
(SUM(?sumDeaths_1until2YR) as ?totalsumDeaths_1until2YR)
(SUM(?sumDeaths_2until5YR) as ?totalsumDeaths_2until5YR)
(SUM(?sumDeaths_5YR) as ?totalsumDeaths_5YR)
```

```
?maindiagnosis

WHERE {
{
?patient a fvc:Patient_hold .
}
UNION
{
{
SELECT
((SUM(?TOTALTIME1YR)/COUNT(?patient)) AS ?personTime_1YR)
((SUM(?TOTALTIME1_2YR)/COUNT(?patient)) AS ?personTime_1until2YR)
((SUM(?TOTALTIME2_5YR)/COUNT(?patient)) AS ?personTime_2until5YR)
((SUM(?TOTALTIMEAFTER5YR)/COUNT(?patient))AS ?personTime_after5YR)

(SUM(?inf1yr) AS ?sumDeaths_1YR)
(SUM(?inf1until2yr) AS ?sumDeaths_1until2YR)
(SUM(?inf2until5yr)AS?sumDeaths_2until5YR)
(SUM(?infafter5yr)AS?sumDeaths_5YR)

?maindiagnosis

WHERE {
?patient a fvc:Patient;
fvc:hasClinicalOutcome ?clinicalOutcome;
fvc:hasPatientOverview ?patientOverview.
?patientOverview fvc:hasDiagnosis ?dia.
?clinicalOutcome fvc:lastRecordedContact ?lastRecordedContact;
fvc:dateOfDeath ?deathDate.
?dia fvc:dateOfDiagnosis ?dateOfDiagnosis.
?dia fvc:mainDiagnosis ?maindiagnosis.

BIND(xsd:integer(xsd:float(str(floor((fn:days-from-duration(?deathDate-
?dateOfDiagnosis)))))) AS ?timeDiagnosisDeath)
BIND(IF(?timeDiagnosisDeath <365.25, 1, 0) AS ?inf1yr)
BIND(IF(?timeDiagnosisDeath <730.5 && ?timeDiagnosisDeath >=365.25, 1, 0) AS
?inf1until2yr)
BIND(IF(?timeDiagnosisDeath <1826.25 && ?timeDiagnosisDeath >=730.5, 1, 0) AS
?inf2until5yr)
BIND(IF(?timeDiagnosisDeath >=1826.25, 1, 0) AS ?infafter5yr)
{
SELECT
(SUM(?personTimeDays_1YR)/365.25 AS ?TOTALTIME1YR)
(SUM(?personTimeDays_1until2YR)/365.25 AS ?TOTALTIME1_2YR)
(SUM(?personTimeDays_2until5YR)/365.25 AS ?TOTALTIME2_5YR)
(SUM(?personTimeDays_after5YR)/365.25 AS ?TOTALTIMEAFTER5YR)
```

```
?maindiagnosis


WHERE {
?patient a fvc:Patient;
fvc:hasClinicalOutcome ?clinicalOutcome;
fvc:hasPatientOverview ?patientOverview.
?clinicalOutcome fvc:lastRecordedContact ?lastRecordedContact;
fvc:death ?hasDied.
?patientOverview fvc:hasDiagnosis ?dia.
?dia fvc:mainDiagnosis ?maindiagnosis.
?dia fvc:dateOfDiagnosis ?dateOfDiagnosis .
OPTIONAL{ ?clinicalOutcome fvc:dateOfDeath ?dateDeath . }
BIND(coalesce(?dateDeath, ?lastRecordedContact) as ?lastfollowup)
BIND("3000-01-01T00:00:00"^^xsd:dateTime as ?defaultDateOfDeath)
BIND(coalesce(?dateDeath, ?defaultDateOfDeath) as ?dateOfDeath)
  BIND(IF(?dateOfDeath < ?lastfollowup, ?dateOfDeath, ?lastfollowup) AS ?lastDate)
BIND(xsd:integer(xsd:float(str(floor((fn:days-from-duration(?lastDate - ?dateOfDiagnosis))))))
AS ?patientTotalTime)
BIND(IF(?patientTotalTime >= 365.25, 365.25, ?patientTotalTime) AS ?personTimeDays_1YR)
BIND(IF(?patientTotalTime >= 730.5, 730.5, ?patientTotalTime) AS ?personTimeDays_2YR)
BIND(IF(?patientTotalTime >= 1826.25, 1826.25, ?patientTotalTime) AS
?personTimeDays_5YR)
BIND(?personTimeDays_2YR - ?personTimeDays_1YR AS ?personTimeDays_1until2YR)
BIND(?personTimeDays_5YR - ?personTimeDays_2YR AS ?personTimeDays_2until5YR)
BIND(?patientTotalTime - ?personTimeDays_5YR AS ?personTimeDays_after5YR)
}
GROUP BY ?maindiagnosis
}
}
GROUP BY ?maindiagnosis
}
}
}
GROUP BY ?maindiagnosis

# Query 12.2b: Mortality-rate. This query returns the follow up time for mortality rates even
if the number of events in one diagnosis group is zero
PREFIX fvc: <http://w3id.org/FAIRVASC#>
PREFIX bvas: <http://w3id.org/BVAS#>
PREFIX xsd: <http://www.w3.org/2001/XMLSchema#>
PREFIX fn:   <http://www.w3.org/2005/xpath-functions#>

SELECT
(SUM(?personTime_1YR) as ?totalpersonTime_1YR)
(SUM(?personTime_1until2YR) as ?totalpersonTime_1until2YR)
(SUM(?personTime_2until5YR) as ?totalpersonTime_2until5YR)
```

```
(SUM(?personTime_after5YR) as ?totalpersonTime_after5YR)

?maindiagnosis

WHERE {
{
?patient a fvc:Patient_hold .
}
UNION
{
{
SELECT
((SUM(?TOTALTIME1YR)/COUNT(?patient)) AS ?personTime_1YR)
((SUM(?TOTALTIME1_2YR)/COUNT(?patient)) AS ?personTime_1until2YR)
((SUM(?TOTALTIME2_5YR)/COUNT(?patient)) AS ?personTime_2until5YR)
((SUM(?TOTALTIMEAFTER5YR)/COUNT(?patient))AS ?personTime_after5YR)

(SUM(?inf1yr) AS ?sumDeaths_1YR)
(SUM(?inf1until2yr) AS ?sumDeaths_1until2YR)
(SUM(?inf2until5yr)AS?sumDeaths_2until5YR)
(SUM(?infafter5yr)AS?sumDeaths_5YR)

?maindiagnosis

WHERE {
?patient a fvc:Patient;
fvc:hasClinicalOutcome ?clinicalOutcome;
fvc:hasPatientOverview ?patientOverview.
?patientOverview fvc:hasDiagnosis ?dia.
?clinicalOutcome fvc:lastRecordedContact ?lastRecordedContact.
    OPTIONAL {?clinicalOutcome fvc:dateOfDeath ?deathDate.}
?dia fvc:dateOfDiagnosis ?dateOfDiagnosis.
?dia fvc:mainDiagnosis ?maindiagnosis.

BIND(xsd:integer(xsd:float(str(floor((fn:days-from-duration(?deathDate-
?dateOfDiagnosis)))))) AS ?timeDiagnosisDeath)
BIND(IF(?timeDiagnosisDeath <365.25, 1, 0) AS ?inf1yr)
BIND(IF(?timeDiagnosisDeath <730.5 && ?timeDiagnosisDeath >=365.25, 1, 0) AS
?inf1until2yr)
BIND(IF(?timeDiagnosisDeath <1826.25 && ?timeDiagnosisDeath >=730.5, 1, 0) AS
?inf2until5yr)
BIND(IF(?timeDiagnosisDeath >=1826.25, 1, 0) AS ?infafter5yr)
{
SELECT
(SUM(?personTimeDays_1YR)/365.25 AS ?TOTALTIME1YR)
(SUM(?personTimeDays_1until2YR)/365.25 AS ?TOTALTIME1_2YR)
(SUM(?personTimeDays_2until5YR)/365.25 AS ?TOTALTIME2_5YR)
```

```
(SUM(?personTimeDays_after5YR)/365.25 AS ?TOTALTIMEAFTER5YR)
?maindiagnosis


WHERE {
?patient a fvc:Patient;
fvc:hasClinicalOutcome ?clinicalOutcome;
fvc:hasPatientOverview ?patientOverview.
?clinicalOutcome fvc:lastRecordedContact ?lastRecordedContact;
fvc:death ?hasDied.
?patientOverview fvc:hasDiagnosis ?dia.
?dia fvc:mainDiagnosis ?maindiagnosis.
?dia fvc:dateOfDiagnosis ?dateOfDiagnosis .
OPTIONAL{ ?clinicalOutcome fvc:dateOfDeath ?dateDeath . }
BIND(coalesce(?dateDeath, ?lastRecordedContact) as ?lastfollowup)
BIND("3000-01-01T00:00:00"^^xsd:dateTime as ?defaultDateOfDeath)
BIND(coalesce(?dateDeath, ?defaultDateOfDeath) as ?dateOfDeath)
  BIND(IF(?dateOfDeath < ?lastfollowup, ?dateOfDeath, ?lastfollowup) AS ?lastDate)
BIND(xsd:integer(xsd:float(str(floor((fn:days-from-duration(?lastDate - ?dateOfDiagnosis))))))
AS ?patientTotalTime)
BIND(IF(?patientTotalTime >= 365.25, 365.25, ?patientTotalTime) AS ?personTimeDays_1YR)
BIND(IF(?patientTotalTime >= 730.5, 730.5, ?patientTotalTime) AS ?personTimeDays_2YR)
BIND(IF(?patientTotalTime >= 1826.25, 1826.25, ?patientTotalTime) AS
?personTimeDays_5YR)
BIND(?personTimeDays_2YR - ?personTimeDays_1YR AS ?personTimeDays_1until2YR)
BIND(?personTimeDays_5YR - ?personTimeDays_2YR AS ?personTimeDays_2until5YR)
BIND(?patientTotalTime - ?personTimeDays_5YR AS ?personTimeDays_after5YR)
}
GROUP BY ?maindiagnosis
}
}
GROUP BY ?maindiagnosis
}
}
}
GROUP BY ?maindiagnosis
```

```
# Query 11.2a: UPDATE. Event-rate of ESKD. This query calculates the number of events of ESKD in four time intervals and the sum of total patient follow-up time in each time frame. End of follow up is date of ESKD if occuring first, if not it is date of death, if not it is date of last visit. It assumes good data quality, no "negative" follow up time etc. All this is stratified per diagnosis. If no event is occurring in any of the timeframes, the diagnosis will not show.

PREFIX fvc: <http://w3id.org/FAIRVASC#>
PREFIX bvas: <http://w3id.org/BVAS#>
```

```
PREFIX xsd: <http://www.w3.org/2001/XMLSchema#>
PREFIX fn:   <http://www.w3.org/2005/xpath-functions#>

SELECT
(SUM(?personTime_1YR) as ?totalpersonTime_1YR)
(SUM(?personTime_1until2YR) as ?totalpersonTime_1until2YR)
(SUM(?personTime_2until5YR) as ?totalpersonTime_2until5YR)
(SUM(?personTime_after5YR) as ?totalpersonTime_after5YR)

(SUM(?sumDeaths_1YR) as ?totalsumESKD_1YR)
(SUM(?sumDeaths_1until2YR) as ?totalsumESKD_1until2YR)
(SUM(?sumDeaths_2until5YR) as ?totalsumESKD_2until5YR)
(SUM(?sumDeaths_5YR) as ?totalsumESKD_5YR)

?maindiagnosis

WHERE {
{
?patient a fvc:Patient_hold .
}
UNION
{
{
SELECT
((SUM(?TOTALTIME1YR)/COUNT(?patient)) AS ?personTime_1YR)
((SUM(?TOTALTIME1_2YR)/COUNT(?patient)) AS ?personTime_1until2YR)
((SUM(?TOTALTIME2_5YR)/COUNT(?patient)) AS ?personTime_2until5YR)
((SUM(?TOTALTIMEAFTER5YR)/COUNT(?patient))AS ?personTime_after5YR)

(SUM(?inf1yr) AS ?sumDeaths_1YR)
(SUM(?inf1until2yr) AS ?sumDeaths_1until2YR)
(SUM(?inf2until5yr)AS?sumDeaths_2until5YR)
(SUM(?infafter5yr)AS?sumDeaths_5YR)

?maindiagnosis

WHERE {
?patient a fvc:Patient;
fvc:hasClinicalOutcome ?clinicalOutcome;
fvc:hasPatientOverview ?patientOverview.
?patientOverview fvc:hasDiagnosis ?dia.
?clinicalOutcome fvc:lastRecordedContact ?lastRecordedContact;
fvc:dateOfESKD ?deathDate.
?dia fvc:dateOfDiagnosis ?dateOfDiagnosis.
?dia fvc:mainDiagnosis ?maindiagnosis.
```

```
BIND(xsd:integer(xsd:float(str(floor((fn:days-from-duration(?deathDate-
?dateOfDiagnosis)))))) AS ?timeDiagnosisDeath)
BIND(IF(?timeDiagnosisDeath <365.25, 1, 0) AS ?inf1yr)
BIND(IF(?timeDiagnosisDeath <730.5 && ?timeDiagnosisDeath >=365.25, 1, 0) AS
?inf1until2yr)
BIND(IF(?timeDiagnosisDeath <1826.25 && ?timeDiagnosisDeath >=730.5, 1, 0) AS
?inf2until5yr)
BIND(IF(?timeDiagnosisDeath >=1826.25, 1, 0) AS ?infafter5yr)
{
SELECT
(SUM(?personTimeDays_1YR)/365.25 AS ?TOTALTIME1YR)
(SUM(?personTimeDays_1until2YR)/365.25 AS ?TOTALTIME1_2YR)
(SUM(?personTimeDays_2until5YR)/365.25 AS ?TOTALTIME2_5YR)
(SUM(?personTimeDays_after5YR)/365.25 AS ?TOTALTIMEAFTER5YR)
?maindiagnosis


WHERE {
?patient a fvc:Patient;
fvc:hasClinicalOutcome ?clinicalOutcome;
fvc:hasPatientOverview ?patientOverview.
?clinicalOutcome fvc:lastRecordedContact ?lastRecordedContact;
fvc:death ?hasDied.
?patientOverview fvc:hasDiagnosis ?dia.
?dia fvc:mainDiagnosis ?maindiagnosis.
?dia fvc:dateOfDiagnosis ?dateOfDiagnosis .
OPTIONAL{ ?clinicalOutcome fvc:dateOfESKD ?dateESKD . }
OPTIONAL{ ?clinicalOutcome fvc:dateOfDeath ?dateDeath . }
BIND("3000-01-01T00:00:00"^^xsd:dateTime as ?defaultDateOfESKD)
BIND(coalesce(?dateESKD, ?defaultDateOfESKD) as ?dateOfESKD)
BIND(coalesce(?dateDeath, ?lastRecordedContact) as ?lastfollowup)
BIND(IF(?dateOfESKD < ?lastfollowup, ?dateOfESKD, ?lastfollowup) AS ?lastDate)
BIND(xsd:integer(xsd:float(str(floor((fn:days-from-duration(?lastDate - ?dateOfDiagnosis))))))
AS ?patientTotalTime)
BIND(IF(?patientTotalTime >= 365.25, 365.25, ?patientTotalTime) AS ?personTimeDays_1YR)
BIND(IF(?patientTotalTime >= 730.5, 730.5, ?patientTotalTime) AS ?personTimeDays_2YR)
BIND(IF(?patientTotalTime >= 1826.25, 1826.25, ?patientTotalTime) AS
?personTimeDays_5YR)
BIND(?personTimeDays_2YR - ?personTimeDays_1YR AS ?personTimeDays_1until2YR)
BIND(?personTimeDays_5YR - ?personTimeDays_2YR AS ?personTimeDays_2until5YR)
BIND(?patientTotalTime - ?personTimeDays_5YR AS ?personTimeDays_after5YR)
}
GROUP BY ?maindiagnosis
}
}
GROUP BY ?maindiagnosis
}
```

```
}
}
GROUP BY ?maindiagnosis


# Query 11.2b: UPDATE. Event-rate of ESKD. This query returns the follow up time even if
there are now event in the diagnosis

PREFIX fvc: <http://w3id.org/FAIRVASC#>
PREFIX bvas: <http://w3id.org/BVAS#>
PREFIX xsd: <http://www.w3.org/2001/XMLSchema#>
PREFIX fn:   <http://www.w3.org/2005/xpath-functions#>

SELECT
(SUM(?personTime_1YR) as ?totalpersonTime_1YR)
(SUM(?personTime_1until2YR) as ?totalpersonTime_1until2YR)
(SUM(?personTime_2until5YR) as ?totalpersonTime_2until5YR)
(SUM(?personTime_after5YR) as ?totalpersonTime_after5YR)

?maindiagnosis

WHERE {
{
?patient a fvc:Patient_hold .
}
UNION
{
{
SELECT
((SUM(?TOTALTIME1YR)/COUNT(?patient)) AS ?personTime_1YR)
((SUM(?TOTALTIME1_2YR)/COUNT(?patient)) AS ?personTime_1until2YR)
((SUM(?TOTALTIME2_5YR)/COUNT(?patient)) AS ?personTime_2until5YR)
((SUM(?TOTALTIMEAFTER5YR)/COUNT(?patient))AS ?personTime_after5YR)

(SUM(?inf1yr) AS ?sumDeaths_1YR)
(SUM(?inf1until2yr) AS ?sumDeaths_1until2YR)
(SUM(?inf2until5yr)AS?sumDeaths_2until5YR)
(SUM(?infafter5yr)AS?sumDeaths_5YR)

?maindiagnosis

WHERE {
?patient a fvc:Patient;
fvc:hasClinicalOutcome ?clinicalOutcome;
fvc:hasPatientOverview ?patientOverview.
?patientOverview fvc:hasDiagnosis ?dia.
?clinicalOutcome fvc:lastRecordedContact ?lastRecordedContact.
```

```
    OPTIONAL {?clinicalOutcome  fvc:dateOfESKD ?deathDate.}
?dia fvc:dateOfDiagnosis ?dateOfDiagnosis.
?dia fvc:mainDiagnosis ?maindiagnosis.

BIND(xsd:integer(xsd:float(str(floor((fn:days-from-duration(?deathDate-
?dateOfDiagnosis)))))) AS ?timeDiagnosisDeath)
BIND(IF(?timeDiagnosisDeath <365.25, 1, 0) AS ?inf1yr)
BIND(IF(?timeDiagnosisDeath <730.5 && ?timeDiagnosisDeath >=365.25, 1, 0) AS
?inf1until2yr)
BIND(IF(?timeDiagnosisDeath <1826.25 && ?timeDiagnosisDeath >=730.5, 1, 0) AS
?inf2until5yr)
BIND(IF(?timeDiagnosisDeath >=1826.25, 1, 0) AS ?infafter5yr)
{
SELECT
(SUM(?personTimeDays_1YR)/365.25 AS ?TOTALTIME1YR)
(SUM(?personTimeDays_1until2YR)/365.25 AS ?TOTALTIME1_2YR)
(SUM(?personTimeDays_2until5YR)/365.25 AS ?TOTALTIME2_5YR)
(SUM(?personTimeDays_after5YR)/365.25 AS ?TOTALTIMEAFTER5YR)
?maindiagnosis


WHERE {
?patient a fvc:Patient;
fvc:hasClinicalOutcome ?clinicalOutcome;
fvc:hasPatientOverview ?patientOverview.
?clinicalOutcome fvc:lastRecordedContact ?lastRecordedContact;
fvc:death ?hasDied.
?patientOverview fvc:hasDiagnosis ?dia.
?dia fvc:mainDiagnosis ?maindiagnosis.
?dia fvc:dateOfDiagnosis ?dateOfDiagnosis .
OPTIONAL{ ?clinicalOutcome fvc:dateOfESKD ?dateESKD . }
OPTIONAL{ ?clinicalOutcome fvc:dateOfDeath ?dateDeath . }
BIND("3000-01-01T00:00:00"^^xsd:dateTime as ?defaultDateOfESKD)
BIND(coalesce(?dateESKD, ?defaultDateOfESKD) as ?dateOfESKD)
BIND(coalesce(?dateDeath, ?lastRecordedContact) as ?lastfollowup)
BIND(IF(?dateOfESKD < ?lastfollowup, ?dateOfESKD, ?lastfollowup) AS ?lastDate)
BIND(xsd:integer(xsd:float(str(floor((fn:days-from-duration(?lastDate - ?dateOfDiagnosis))))))
AS ?patientTotalTime)
BIND(IF(?patientTotalTime >= 365.25, 365.25, ?patientTotalTime) AS ?personTimeDays_1YR)
BIND(IF(?patientTotalTime >= 730.5, 730.5, ?patientTotalTime) AS ?personTimeDays_2YR)
BIND(IF(?patientTotalTime >= 1826.25, 1826.25, ?patientTotalTime) AS
?personTimeDays_5YR)
BIND(?personTimeDays_2YR - ?personTimeDays_1YR AS ?personTimeDays_1until2YR)
BIND(?personTimeDays_5YR - ?personTimeDays_2YR AS ?personTimeDays_2until5YR)
BIND(?patientTotalTime - ?personTimeDays_5YR AS ?personTimeDays_after5YR)
}
GROUP BY ?maindiagnosis
```

```
}
}
GROUP BY ?maindiagnosis
}
}
}
GROUP BY ?maindiagnosis


# Query 10a: Mean s-creatinine at diagnosis (Czech special)
PREFIX fvc: <http://w3id.org/FAIRVASC#>
PREFIX bvas: <http://w3id.org/BVAS#>

SELECT
(AVG(?crea) AS ?avg_crea)

WHERE {
?patient a fvc:Patient.
?patient fvc:hasPatientOverview ?PatientOverview.
?PatientOverview fvc:hasCreatinineAtDiagnosis ?creadiagnosis.
?creadiagnosis fvc:testValue ?crea.
  FILTER(?crea != "1800"^^<http://www.w3.org/2001/XMLSchema#double>)

}



# Query 10b: Mean s-creatinine at diagnosis availability (Czech special)

PREFIX fvc: <http://w3id.org/FAIRVASC#>
PREFIX bvas: <http://w3id.org/BVAS#>

SELECT (COUNT(DISTINCT ?patient) AS ?creadiagnosis_n)

WHERE {
?patient a fvc:Patient.
?patient fvc:hasPatientOverview ?PatientOverview.
?PatientOverview fvc:hasCreatinineAtDiagnosis ?creadiagnosis.
?creadiagnosis fvc:testValue ?crea.
  FILTER(?crea != "1800"^^<http://www.w3.org/2001/XMLSchema#double>)

}


# Query 10b.2: Mean s-creatinine at diagnosis availability excluded because of dialysis
(Czech special)
```

```
PREFIX fvc: <http://w3id.org/FAIRVASC#>
PREFIX bvas: <http://w3id.org/BVAS#>

SELECT (COUNT(DISTINCT ?patient) AS ?creadiagnosis_n)

WHERE {
?patient a fvc:Patient.
?patient fvc:hasPatientOverview ?PatientOverview.
?PatientOverview fvc:hasCreatinineAtDiagnosis ?creadiagnosis.
?creadiagnosis fvc:testValue ?crea.
  FILTER(?crea = "1800"^^<http://www.w3.org/2001/XMLSchema#double>)
}
```

```
# Query 10c: Creatinine at diagnosis variance in registry (this has to be sqrt to retrieve standard deviation (SD) of age which can't be done in pure SPARQL) (Czech special)

PREFIX fvc: <http://w3id.org/FAIRVASC#>
PREFIX bvas: <http://w3id.org/BVAS#>


SELECT(SUM((?crea-?avg_crea)*(?crea-?avg_crea))/(COUNT(?creadiagnosis) - 1) as
?variance)

WHERE {
?patient a fvc:Patient.
?patient fvc:hasPatientOverview ?PatientOverview.
?PatientOverview fvc:hasCreatinineAtDiagnosis ?creadiagnosis.
?creadiagnosis fvc:testValue ?crea.
 FILTER(?crea != "1800"^^<http://www.w3.org/2001/XMLSchema#double>)
 {

SELECT
(AVG(?crea) AS ?avg_crea)

WHERE {
?patient a fvc:Patient.
?patient fvc:hasPatientOverview ?PatientOverview.
?PatientOverview fvc:hasCreatinineAtDiagnosis ?creadiagnosis.
?creadiagnosis fvc:testValue ?crea.
      FILTER(?crea != "1800"^^<http://www.w3.org/2001/XMLSchema#double>)
```

Supplemental table 4

Table 4. Data quality percentages of key variables per registry

	Czech	FVSG	GeVas	POLVAS	RKD	Skåne
Uniqueness						
Number of IDs, n (%)	311 (100)	2804 (100)	165 (100)	987 (100)	663 (100)	374 (100)
Duplicate IDs, n (%)	0 (0)	0 (0)	0 (0)	0 (0)	0 (0)	0 (0)
Potential duplicate entries, n (%) [a]	0 (0)	0 (0)	0 (0)	22 (2.2)	0 (0)	0 (0)
Consistency [b]						
Gender, n (%)	311 (100)	2804 (100)	165 (100)	987 (100)	663 (100)	374 (100)
Date of birth, n (%)	311 (100)	2667 (100)	165 (100)	987 (100)	661 (100)	374 (100)
ELISA ANCA,* n (%)	310 (100)	2219 (100)	164 (100)	973 (100)	663 (100)	374 (100)
BVAS*, n (%)	154 (100)	1817 (100)	162 (100)	NA	429 (100)	374 (100)
S-creatinine, n (%)	226 (100)	2017 (100)	135 (100)	936 (100)	470 (100)	371 (100)
C-reactive protein, n (%)	154 (100)	1472 (100)	157 (100)	860 (100)	389 (100)	364 (100)
Induction treatment, n (%)	NA	NA	160 (100)	965 (100)	656 (100)	374 (100)
Date of death, n (%)	58 (100)	330 (100)	3 (100)	110 (100)	123 (100)	186 (100)
Date of ESKD, n (%)	37 (100)	297 (100)	5 (100)	NA	126 (100)	55 (100)
Plausibility						
Date of death >= date of birth, n (%)	58 (100)	320 (96.7)	3 (100)	110 (100)	123 (100)	186 (100)

Date of death >= date of diagnosis, n (%)	58 (100)	309 (93.6)	3 (100)	108 (98.2)	123 (100)	186 (100)
BVAS, n (%)	154 (100)	1817 (100)	162 (100)	NA	429 (100)	374 (100)
S-creatinine, n (%)	226 (100)	2015 (99.9)	135 (100)	936 (100)	470 (100)	371 (100)
C-reactive protein, n (%)	154 (100)	1472 (100)	155 (98.7)	859 (99.8)	389 (100)	364 (100)
Completeness						
Gender, n (%)	311 (100)	2804 (100)	165 (100)	987 (100)	663 (100)	374 (100)
Date of birth, n (%)	311 (100)	2667 (95.1)	165 (100)	987 (100)	661 (99.9)	374 (100)
ELISA ANCA, n (%)	310 (99.7)	2219 (79.1)	164 (99.4)	973 (98.6)	663 (100)	374 (100)
BVAS, n (%)	154 (49.5)	1817 (64.8)	162 (98.2)	NA	429 (64.7)	374 (100)
S-creatinine, n (%)	226 (72.7)	2017 (71.2)	135 (81.8)	936 (94.8)	470 (70.9)	371 (99.2)
C-reactive protein, n (%)	154 (49.5)	1472 (53.2)	157 (95.2)	860 (87.1)	389 (58.7)	364 (97.3)
Induction treatment, n (%)	NA	NA	160 (96.9)	975 (98.8)	656 (98.9)	374 (100)
Date of death [c], n (%)	58 (100)	330 (94.0)	3 (75.0)	110 (96.5)	123 (99.2)	186 (99.5)
Date of ESKD , n (%)	37 (100)	297 (100)	5 (100)	NA	126 (100)	55 (100)
Correctness						
Gender, n (%)	10 (100)	NA	9 (100)	10 (100)	10 (100)	10 (100)
Date of birth, n (%)	10 (100)	NA	9 (100)	9 (90.0)	10 (100)	10 (100)
ELISA ANCA, n (%)	10 (100)	NA	9 (100)	10 (100)	10 (100)	10 (100)
BVAS, n (%)	10 (100)	NA	9 (100)	NA	6 (60.0)	10 (100)
S-creatinine, n (%)	10 (100)	NA	9 (100)	10 (100)	8 (80.0)	10 (100)
C-reactive protein, n (%)	10 (100)	NA	7 (77.8)	9 (90.0)	9 (90.0)	9 (90.0)

Induction treatment, n (%)	NA	NA	9 (100)	10 (100)	10 (100)	8 (80.0)
Date of death, n (%)	10 (100)	NA	9 (100)	NA	NA	10 (100)
Date of ESKD, n (%)	10 (100)	NA	NA	NA	NA	10 (100)

[a] Potential duplicate entries are cases sharing date of birth, gender and date of diagnosis or date of death. [b] Consistency percentages are calculated on complete data. [c] Percentage calculated over patients that have died: 58 (Czech), 4 (Gevas), 351 (FVSG), 114 (POLVAS), 124 (RKD), 187 (Skåne). ANCA = Anti-neutrophil cytoplasmic antibody. BVAS = Birmingham vasculitis activity score version 3. ESKD = end-stage kidney disease.
